# Mechanisms affecting the gut of preterm infants in enteral feeding trials: a nested cohort within a randomised controlled trial of lactoferrin

**DOI:** 10.1136/archdischild-2022-324477

**Published:** 2022-11-17

**Authors:** Greg Young, Janet E Berrington, Stephen Cummings, Jon Dorling, Andrew K Ewer, Alessandra Frau, Lauren Lett, Chris Probert, Ed Juszczak, John Kirby, Lauren C Beck, Victoria L Renwick, Christopher Lamb, Clare V Lanyon, William McGuire, Christopher Stewart, Nicholas Embleton

**Affiliations:** 1 Applied Sciences, Northumbria University Faculty of Health and Life Sciences, Newcastle upon Tyne, England, UK; 2 Microbial Environments, Hub for Biotechnology in the Built Environment, Newcastle upon Tyne, England, UK; 3 Translational and Clinical Research Institute, Newcastle University, Newcastle upon Tyne, UK; 4 Newcastle Neonatal Service, Ward 35 Neonatal Unit, Royal Victoria Infirmary, Newcastle Upon Tyne Hospitals NHS Foundation Trust, Newcastle Upon Tyne, UK; 5 School of Health and Life Sciences, Teesside University, Middlesbrough, North Yorkshire, UK; 6 Department of Neonatal Medicine, University Hospital Southampton NHS Foundation Trust, Southampton, UK; 7 Institute of Metabolism and Systems Research, University of Birmingham, Birmingham, UK; 8 Gastroenterology Research Unit, Institute of Translational Medicine, University of Liverpool, Liverpool, UK; 9 School of Medicine, University of Nottingham School of Medicine, Nottingham, Notts, UK; 10 Centre for Reviews and Dissemination, University of York, York, North Yorkshire, UK; 11 Population Health Sciences Institute, Newcastle University, Newcastle upon Tyne, UK

**Keywords:** Intensive Care Units, Neonatal, Microbiology, Molecular Biology, Neonatology, Sepsis

## Abstract

**Objective:**

To determine the impact of supplemental bovine lactoferrin on the gut microbiome and metabolome of preterm infants.

**Design:**

Cohort study nested within a randomised controlled trial (RCT). Infants across different trial arms were matched on several clinical variables. Bacteria and metabolite compositions of longitudinal stool and urine samples were analysed to investigate the impact of lactoferrin supplementation.

**Setting:**

Thirteen UK hospitals participating in a RCT of lactoferrin.

**Patients:**

479 infants born <32 weeks’ gestation between June 2016 and September 2017.

**Results:**

10 990 stool and 22 341 urine samples were collected. Analyses of gut microbiome (1304 stools, 201 infants), metabolites (171 stools, 83 infants; 225 urines, 90 infants) and volatile organic compounds (314 stools, 117 infants) were performed. Gut microbiome Shannon diversity at 34 weeks corrected age was not significantly different between infants in the lactoferrin (mean=1.24) or placebo (mean=1.06) groups (p=0.11). Lactoferrin receipt explained less than 1% variance in microbiome compositions between groups. Metabolomic analysis identified six discriminative features between trial groups. Hospital site (16%) and postnatal age (6%) explained the greatest variation in microbiome composition.

**Conclusions:**

This multiomic study identified minimal impacts of lactoferrin but much larger impacts of hospital site and postnatal age. This may be due to the specific lactoferrin product used, but more likely supports the findings of the RCT in which this study was nested, which showed no impact of lactoferrin on reducing rates of sepsis. Multisite mechanistic studies nested within RCTs are feasible and help inform trial interpretation and future trial design.

**WHAT IS ALREADY KNOWN ON THIS TOPIC**

- Gut microbial patterns and function are associated with development of late onset sepsis.
- Lactoferrin supplementation may reduce sepsis but the mechanisms of action in preterm neonates are not clear.
- Lactoferrin has impacts on bacteria and endothelial function in vitro.

**WHAT THIS STUDY ADDS**

- Supplemental bovine lactoferrin has minimal impact on gut microbiome or metabolome in preterm infants.
- Neonatal Intensive Care Unit (NICU)site and postnatal age have a large impact on gut microbial patterns.
- Mechanistic work adds value to clinical trial interpretation and future trial design.

**HOW THIS STUDY MIGHT AFFECT RESEARCH, PRACTICE OR POLICY**

- Embedded mechanistic studies in randomised controlled trials (RCTs) generate important learning. Future neonatal RCTs should consider such approaches.
- The importance of NICU site on the neonatal microbiome and metabolome should be recognised in future related work.
- The dosing and timing schedule employed here demonstrates a lack of impact of enteral bovine lactoferrin.

## Introduction

Globally, around 1.6% of births are at <32 weeks’ gestation (very preterm infant (VPTI)).^1^ Despite increased survival, death and disability due to late onset sepsis (LOS, >72 hours of age) and necrotising enterocolitis (NEC) have increased.^2^ Around 25%–50% of VPTI develop LOS,^3^ and around 5%–7% develop NEC^4^ together contributing to 20%–30% of all preterm deaths.^2 5^

Mother’s own breast milk (MOM) is associated with lower rates of LOS, NEC and mortality^6^; however, potential mechanisms of protection remain poorly understood. Specific proteins, sugars and other ‘bionutrients’ present in MOM are hypothesised to alter the gut microbiome,^7 8^ immune function and directly affect gut endothelium.^9^

Lactoferrin is an abundant milk glycoprotein, present in high concentrations in colostrum^10^ and is proposed as a protective factor against LOS and NEC, with evidence suggesting that it modulates gut microbiota.^11 12^ In vitro studies demonstrate that lactoferrin promotes growth of microbes associated with gut health such as *Bifidobacterium longum* 13 and inhibit bacteria associated with LOS such as *Staphylococcus aureus*.^14^ Iron sequestration by lactoferrin appears key to microbial modulation,^15 16^ with iron-depleted lactoferrin showing different microbial interactions to the iron saturated form. Further protective effects of lactoferrin may arise from interaction with endothelial cells by promoting crypt cell formation^9 17 18^ and attenuating microbial mediated endothelial barrier dysfunction.^19^

In 2017, meta-analysis of randomised control trials (RCTs) of lactoferrin supplementation included six trials (886 participants) and gave a number needed to treat to prevent one case of LOS of 17 (95% CI 10 to 50), but quality was evaluated as low.^20^

The ELFIN trial (Enteral Lactoferrin In Neonates) assessed the clinical impact of supplemental bovine lactoferrin (150 mg/kg/day) compared with placebo, until 34 weeks postmenstrual age,^21 22^ in a multicentre double-blinded RCT of 2203 infants. We nested MAGPIE (Mechanisms Affecting the Gut of Preterm Infants in Enteral feeding trials) within ELFIN to explore potential mechanisms of action of lactoferrin that might improve outcomes in preterm infants.^23^

## Aim

This study aimed to determine the impact of supplemental enteral bovine lactoferrin on gut microbial community structure and function using targeted bacterial sequencing, gas chromatography-mass spectrometry (GC-MS) and liquid chromatography-mass spectrometry (LC-MS). Full details of the study protocol and methodology have been previously reported.^24^

## Methods

### Design and population

Parents of preterm infants <32 weeks’ gestation who were enrolled in the ELFIN study^21 22^ at 13 participating Neonatal Intensive Care Units (NICUs) in England (online supplemental file 1, p2) were offered a parent information sheet.^24^ We obtained signed consent to collect stool and urine from their baby along with permission to store residual samples in an Human Tissue Authority approved biobank (North East Newcastle and North Tyneside 1;21/NE/0024). We gained consent from the National Perinatal Epidemiology Unit Clinical Trials Unit (NPEU CTU) to share anonymised ELFIN trial clinical data with MAGPIE. Demographic and clinical outcome data used were those collected for the ELFIN study,^22^ verified and reviewed by NPEU and at blinded-end-point-review committees. Additional data included daily milk type (breast, formula or mixed) and ELFIN Investigational Medicinal Product (IMP) administration and information on antibiotic and antifungal drug use recorded at sites (online supplemental methods).

10.1136/fetalneonatal-2022-324477.supp1 Supplementary data

10.1136/fetalneonatal-2022-324477.supp2 Supplementary data

MAGPIE was approved by East Midlands – Nottingham 2 Research Ethics Committee (16/EM/0042) and registered prospectively (ISRCTN12554594).

### Sample collection, storage and transport

Daily stool and urine samples were dated, anonymised and analysed with standard operating procedures^24–26^ (online supplemental file 2).

### Sample selection

Infants were categorised as healthy (no NEC or culture positive LOS), NEC or culture positive LOS using ELFIN criteria (online supplemental file 2). Infants with good longitudinal sampling (availability at 0–6 days of life (DOL), 7–9 (DOL), 10–14 (DOL), 20–27 (DOL) and 30–60 (DOL)) were selected from both trial arms and matched for site and gestation wherever possible. Infants from the same multiple pregnancy but allocated to different trial groups were preferentially chosen as optimal matches.

### Analyses

Bacterial communities and metabolite composition of samples were assessed using established, quality controlled, standardised workflows interrogating validated databases as previously reported.^24^ All stool samples contributed to microbiomic analysis via targeted 16S rRNA gene sequencing. Where sample volume and funding allowed, we also performed LC-MS detection of untargeted metabolites and GC-MS detection of volatile organic compounds (VOCs). Blinding to trial arm designation was maintained until all analyses were complete.

### Statistics

Categorical and continuous metadata variables were compared by Fisher’s exact and Kruskal-Wallis test, respectively. Longitudinal analysis was restricted to one sample per timepoint per subject using first available where multiple samples existed. Alpha diversity was assessed by feature richness and Shannon diversity. Feature richness was calculated as the total number of individual features identified within a sample following normalisation. Beta diversity was assessed by weighted Bray-Curtis dissimilarity for bacterial communities and Canberra compositional dissimilarity for LC-MS and VOC metabolite datasets.

Results of previous research by this group was used to determine which clinical variables to include in statistical models.^7 23 24^ General linear mixed models were used to assess the impact of clinical variables on alpha diversity measures. Gestational age, birth weight, milk type at the time of sample, health status (healthy, NEC or LOS), IMP (lactoferrin or placebo) receipt at time of sample, day of life and NICU site were included as fixed effects. Infant identity was included as a random effect. Permutational analysis of variance was used to assess impact of these variables on sample compositions. Microbiome Multivariable Association with Linear Models (MaAsLin2) were used to identify features that differed with lactoferrin receipt (ie, between ELFIN trial arms) both within individual NICUs and across all NICUs. For software packages used, data availability and more detailed descriptions of data transformations or statistical tests, see online supplemental file 2.

## Results

### Infants and samples

About 479 infants <32 weeks’ gestation were recruited from 13 NICUs, and 467 provided usable samples totalling 10 990 stool and 22 341 urine samples. Sampling by site varied with individual NICUs contributing 3 to 34 infants and between 11 and 205 samples (samples collected per NICU site (figure), online supplemental file 1, p3; longitudinal samples received by NICU site (figure and table), online supplemental file 1, p4). MAGPIE infants overall and in each type of subanalysis were comparable with the whole ELFIN cohort (table 1).

**Table 1:** Demographic information for study cohorts and for the whole ELFIN and MAGPIE cohorts

		16S cohort			VOC cohort			LC-MS stool cohort			LC-MS urine cohort			ELFIN cohort			MAGPIE cohort
		**Lactoferrin**	**Placebo**	**P value**	**Lactoferrin**	**Placebo**	**P value**	**Lactoferrin**	**Placebo**	**P value**	**Lactoferrin**	**Placebo**	**P value**	**Lactoferrin**	**Placebo**	Mean (SD)	**All (479)**
Median (IQR)	Gestational age (weeks)	27	27	0.68	28	28	0.85	27	27	0.72	27	27	0.94	29	29		28.4 mean
		(*25–29*)	(*25–29*)		(*26–27*)	(*26–27*)		(*25–29*)	(*25–29*)		(*25–29*)	(*25–29*)		(*27–30*)	(*27–30*)		(*2.3*)
	Birth weight (g)	970	961	0.94	1040	1011	0.52	1006	925	0.95	908	910	0.93	1125.9	1143.3		1120 mean
		*(760–1188*)	*(716–1220*)		*(800–1260*)	*(864–1249*)		*(725–1168*)	*(692–1220*)		*(714–1160*)	*(697–1180*)		*356.2 SD*	*367.1 SD*		(*358*)
	Sample day of life (used in analysis)	19	16	0.83	15	13	0.88	22	19	0.47	21	19	0.38	–	–		–
		(*10–31*)	(*9–30*)		(*8–18*)	(*9–18*)		(*12–31*)	(*12–28*)		(*13–31*)	(*12–28*)		–	–		–
	First IMP exposure (day of life)	5	5	0.53	4	5	0.5	5	5	0.79	5	5	0.8	–	–		–
		(*4–7*)	(*4–6*)		(*4–6*)	(*4–7*)		(*3–7*)	(*4–7*)		(*4–7*)	(*4–8*)		–	–		–
	First feed (day of life)	5	5	0.43	5	5	0.74	5	5	0.92	5	5	0.78	–	–		–
		(*4–8*)	(*4–6*)		(*4–6*)	(*4–7*)		(*3–7*)	(*4–7*)		(*4–7*)	(*4–8*)		–	–		–
	Last parenteral nutrition (day of life)	10	10	0.28	10	9	0.09	10	10	0.17	10	10	0.32	–	–		–
		(*8–15*)	(*6–15*)		(*8–16*)	(*5–13*)		(*9–15*)	(*7–13*)		(*8–16*)	(*6–13*)		–	–		–
Total count	Infants (samples)	98 (620)	103 (629)	–	60 (154)	58 (160)	–	40 (82)	43 (89)	–	41 (100)	49 (125)	–	1098	1099	Total count	479 (33331)
	No disease	57	59	0.89	38	32	0.46	28	24	0.26	27	25	0.15	–	–		–
	Disease (NEC or LOS)	41	44	0.4	22	26	0.46	12	19	0.27	14	24	0.68	–	–		–
	Confirmed NEC	9	14	0.38	7	14	0.09	6	8	0.77	6	13	0.21	63 (6%)	56 (5%)		31 (6.5%)
	Confirmed LOS	32	30	0.65	20	16	0.56	10	11	1	14	15	0.82	190 (17%)	180 (17%)		81 (17%)
	Multiple birth	50	42	0.26	41	28	0.04	19	19	0.83	21	21	0.41	350 (32%)	346 (31%)		145 (30.2%)
	Exclusively maternal milk fed	54	56	0.88	31	30	1	19	24	0.51	21	30	0.5	–	–		–
	Exclusively formula fed	9	11	0.82	7	9	0.7	2	4	0.68	2	4	0.83	–	–		–
	Mixed feeds	35	37	1	23	19	0.6	19	15	0.27	19	16	0.28	–	–		–

IMP, Investigational Medicinal Product; LC-MS, liquid chromatography-mass spectrometry; LOS, late onset sepsis; NEC, necrotising enterocolitis; VOC, volatile organic compound.

Microbiome analysis used 1304 stool samples from 201 infants from all 13 NICUs. LC-MS metabolomics was undertaken on 171 stools from 83 of those infants and 225 urine samples from 90 of those infants. GC-MS VOC analysis was undertaken on 314 stools from 117 of those infants. GC-MS and LC-MS analyses were on samples from the same/next day of life as microbiome samples. Median gestational age at birth of all analysed MAGPIE infants was 27 weeks (IQR 25–29), median birth weight was 965 g (IQR 740–1014). Feeding, antibiotic and probiotic administration practices varied across NICUs, but most (90%) infants received some MOM. Three sites (A, D and E) routinely administered probiotics. In keeping with the ELFIN trial findings, no significant differences in disease prevalence, multiple birth, gestational age, MOM receipt or birth weight were observed between infants receiving lactoferrin supplements or placebo in MAGPIE (table 1).^24^

#### Overall sample composition

About 874 bacterial genera, 36 VOCs and 7457 metabolite features were identified in stool and 7907 metabolite features in urine samples. *Staphylococcus* and *Escherichia* were the dominant genera in the first postnatal days, succeeded by increased anaerobes (Enterobacteriaceae, *Veillonella* and *Bifidobacterium*) in later samples (overall sample compositions over time (figure), online supplemental file 3, p3). Hexanal was the most prevalent VOC (91%), followed by acetic acid (89%) which was also the most prevalent short chain fatty acid (prevalence of VOC features identified (table), online supplemental file 3, p4).

10.1136/fetalneonatal-2022-324477.supp3 Supplementary data

#### Supplemental lactoferrin has a limited impact on gut microbial and metabolite composition

The impact of supplemental lactoferrin versus placebo was assessed using mixed-effects models incorporating available clinical variables. No significant difference was observed in bacterial Shannon diversity between infants receiving lactoferrin (mean 1.24) or placebo (mean 1.06) at 34 weeks corrected age (p=0.11) or across all timepoints (p=0.53) (corrected alpha diversity comparisons per individual analysis mode (table), online supplemental file 3, p5). Likewise, VOC and stool metabolite composition and urinary metabolite composition were unaffected by lactoferrin supplementation (corrected alpha diversity comparisons per individual analysis mode (table), online supplemental file 3, p5).

#### NICU site and infant age at sampling drive gut microbial and metabolite composition

**Figure 1:**
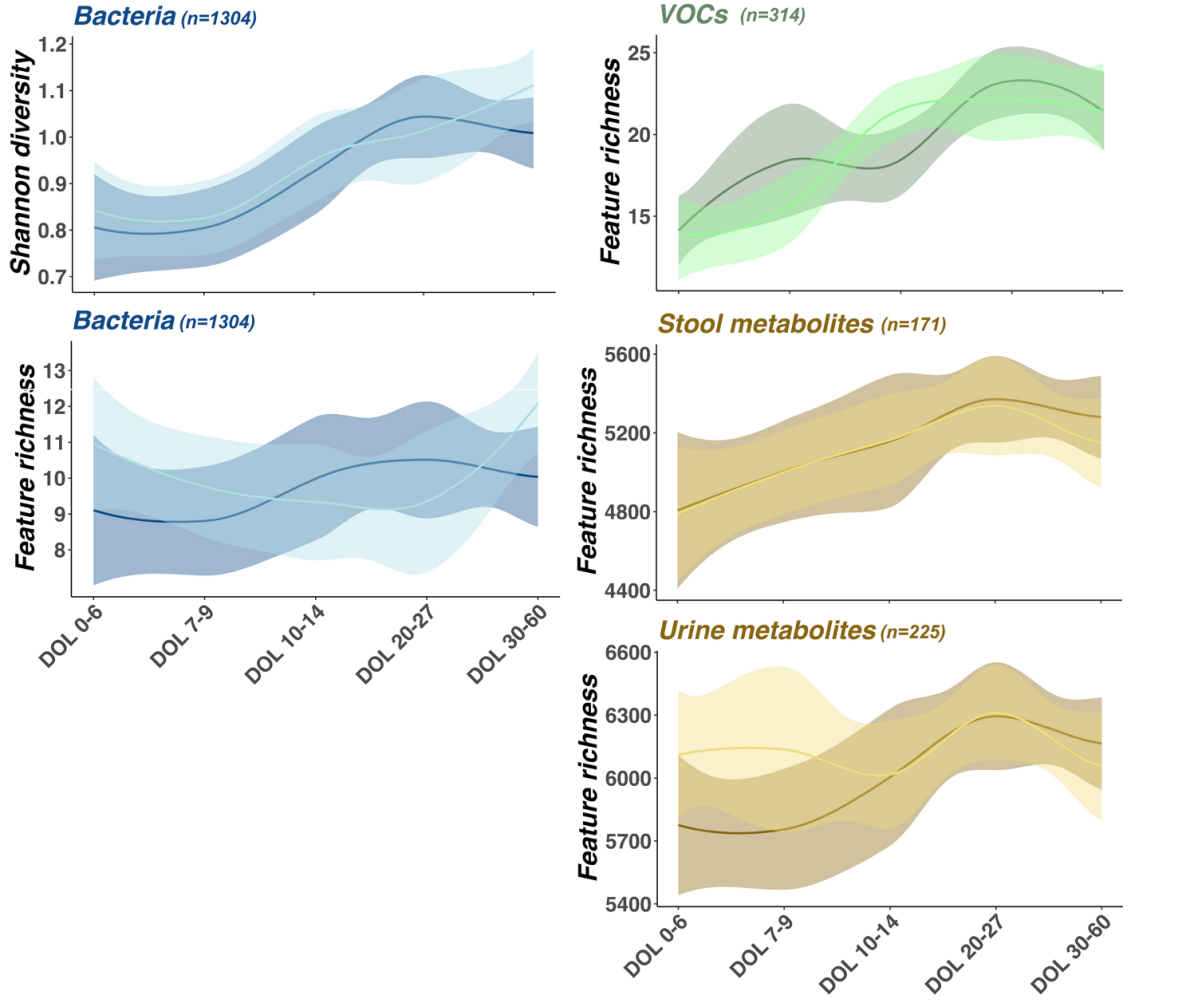
Line chart illustrating alpha diversity of stool bacterial communities (blue), stool volatile organic compounds (VOCs) (green), and stool and urine metabolites (gold) on the y-axis, correlated with day of life (DOL) of infants on the x-axis. Lines on each panel represent mean alpha diversity observed in lactoferrin (dark) and placebo (light) cohorts. Shaded areas around each line represent 95% CIs. Numbers of samples included in each analysis are indicated in plot header (n).

After adjusting for covariates, infant DOL at sampling had the greatest impact on alpha diversity with significant increases in VOC and metabolite richness (p<0.03) as well as bacterial richness (p=0.03) and Shannon diversity (p<0.001) observed over time (figure 1). Greater birth weight was significantly associated with increased bacterial Shannon diversity (p=0.02) and healthy infants had significantly lower stool metabolite richness (p=0.02) (corrected alpha diversity comparisons per individual analysis mode (table), online supplemental file 3, p5).

**Figure 2:**
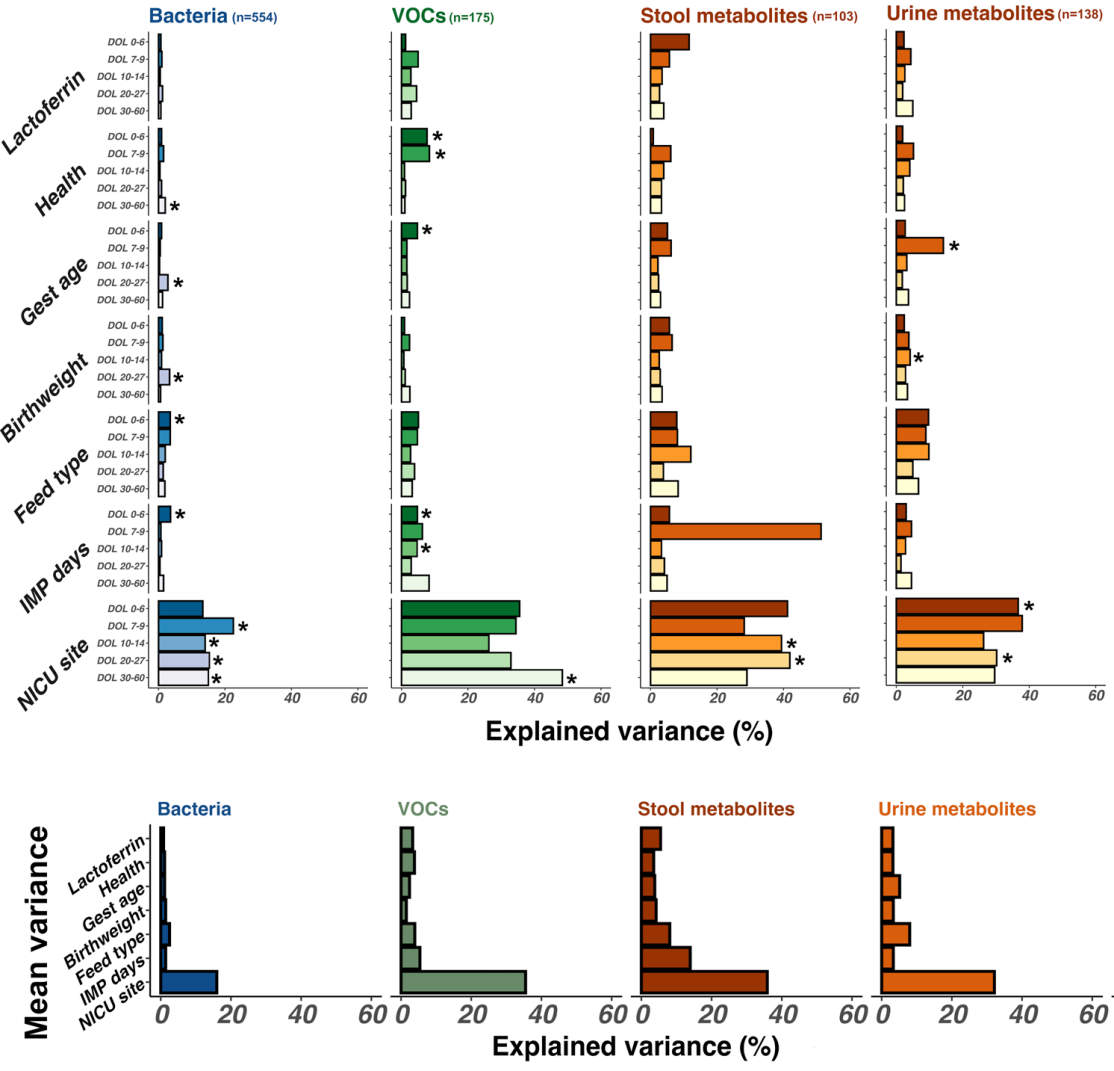
Bar chart demonstrating the impact of clinical variables (grouped on the y-axis) on stool bacterial (blue), stool volatile organic compound (VOC) (green), and stool and urine metabolite composition (gold). Impact is illustrated as explained variance (x-axis). Variables exerting greater influence on sample composition thus have larger bars. Significant associations (p<0.05) are highlighted with an asterisk. One sample per patient per timepoint was included in longitudinal analysis from earliest days of life (DOL) samples (darkest colour) to latest DOL samples (lightest colour). Numbers of samples included in each analysis are indicated in plot header (n). Further details of samples included per timepoint are available in samples per timepoint (figure), online supplemental file 1, p6.

Lactoferrin exposure explained a mean of 3% of the variance between gut microbiota and metabolite sample compositions across all timepoints. NICU site had the greatest influence, describing a mean 30% variance across all timepoints (figure 2). From DOL 7, NICU site had a significant impact on bacterial community composition explaining a mean 16.7% total variance between samples (p<0.01). Before DOL 7, milk type (p=0.03) had the greatest impact on bacterial community composition explaining 3.5% total variance. NICU site influenced stool metabolites at later timepoints: DOL 10–14 and DOL 20–27 (p<0.01); explaining a mean 40% of variance and VOC composition during DOL 0–6 (35.5% total variance; p=0.005) (corrected beta diversity comparisons per individual analysis mode (table), online supplemental file 3, p6).

Despite the lack of overall impact of lactoferrin supplementation, analysis with MaAsLin2 identified some bacterial genera and metabolite features significantly associated with clinical features, including lactoferrin exposure. Models were built to control for clinical covariates including infant age at sampling, health status, feed type, birth weight, gestational age and days of exposure to trial intervention (lactoferrin or placebo). Due to the significant impact of NICU site on sample compositions (figure 2), we initially performed differential feature analysis within each NICU individually.

**Figure 3:**
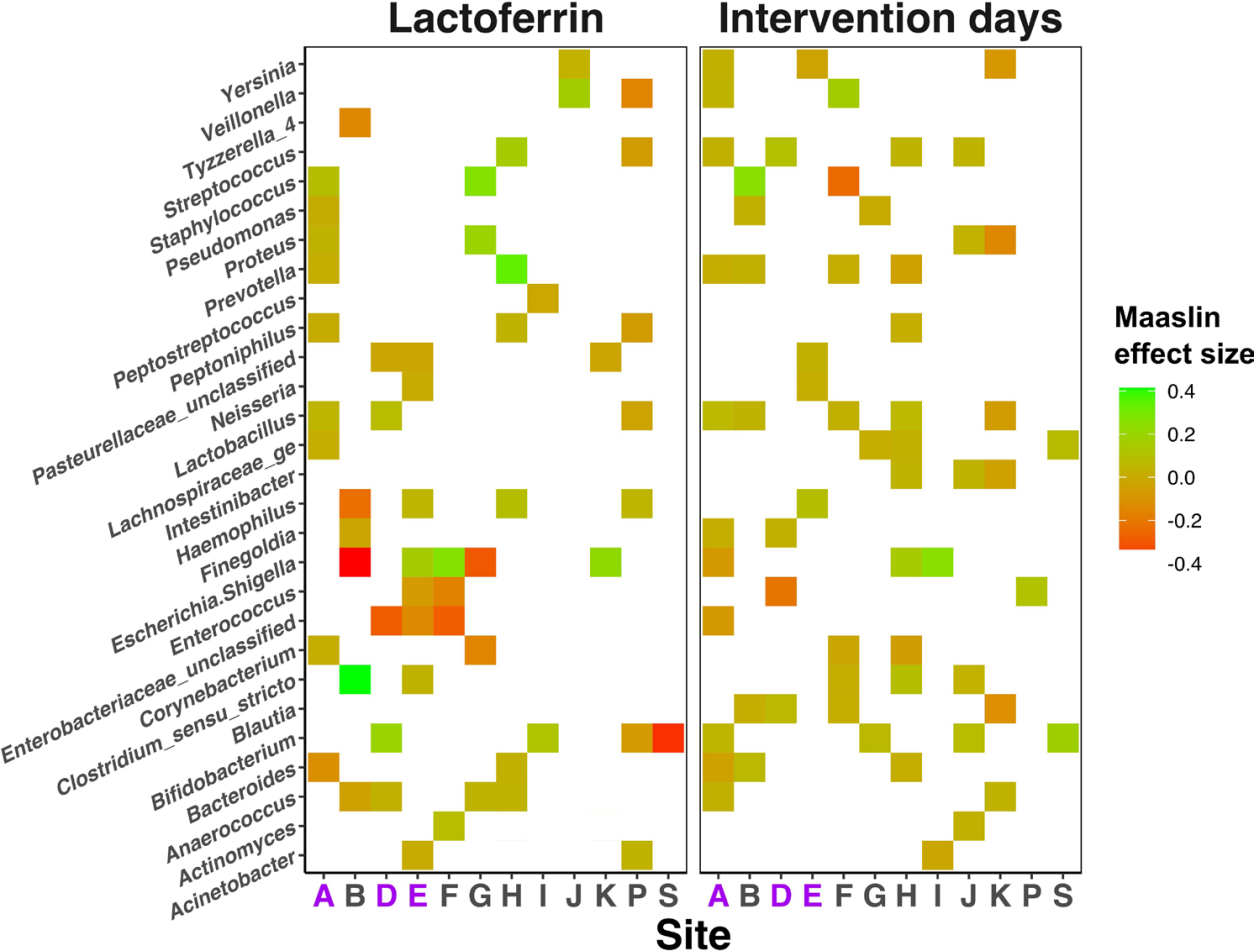
Heatmap showing the impact of lactoferrin supplementation on relative abundance of bacterial genera (y-axis) across each individual Neonatal Intensive Care Unit site (x-axis). Colour is used to illustrate positive (green) and negative (red) associations with either lactoferrin receipt (left panel) or increasing intervention days (right panel). Null associations remain blank. NICU sites administering probiotics are highlighted purple on the x-axis.

Relative abundances of several (19) bacterial and metabolite (1664 stool, 321 urine) features were significantly different between trial arms (figure 3) at many sites, but few were consistent across all NICUs (Sitewise MaAsLin results: number of significantly discriminant features between lactoferrin and placebo samples in each analysis mode (table), online supplemental file 3, p7). *Lactobacillus* was significantly reduced in samples from lactoferrin receiving infants in probiotic administering NICUs A and D (p<0.02, Q<0.12) and *Staphylococcus* was significantly reduced in lactoferrin receiving infants in NICU A (p<0.001, Q<0.001) (figure 3).

**Figure 4:**
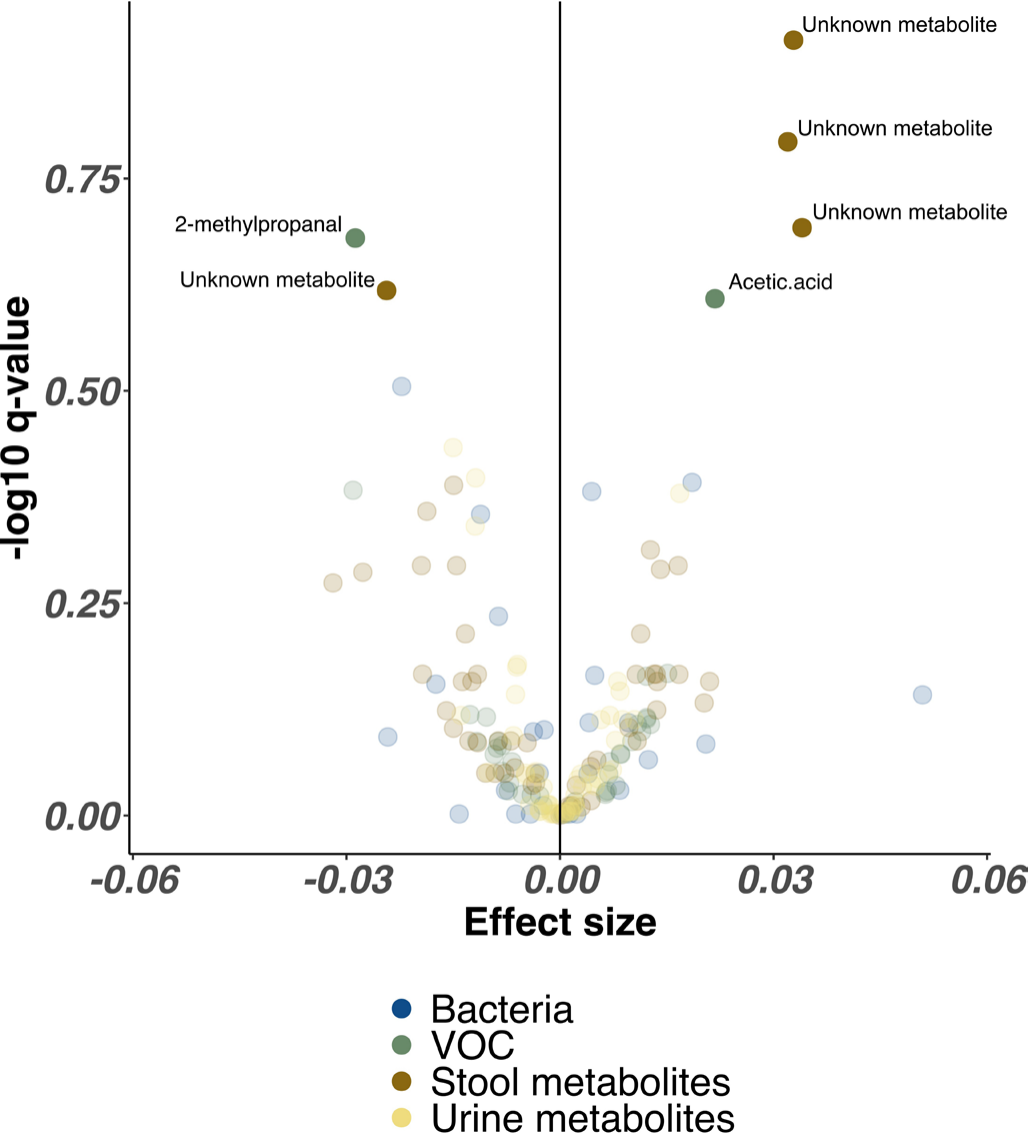
Volcano plot illustrating combined bacterial, volatile organic compound (VOC) and metabolite features associated with lactoferrin supplementation across all Neonatal Intensive Care Unit sites. Each point represents a single feature, coloured by omics method. Higher effect sizes represent greater relative abundance of each feature in lactoferrin receipt cohort, lower effect sizes represent reduced relative abundance of each feature in lactoferrin receipt cohort. Features with significantly altered relative abundance identified by MaAsLin2 (following Benjamini-Hochberg correction) are highlighted with greater opacity and labelled with feature names where available.

In combined analysis from every NICU site controlling for all clinical factors, proportional abundance of only three unidentified stool metabolites (p>0.005, Q>0.20) and acetic acid (p=0.02, Q=0.25) were significantly higher in infants receiving lactoferrin, while one single unidentified stool metabolite (p=0.006, Q=0.24) and 2-methyl-propanal (p=0.01, Q=0.21) were significantly higher in placebo. No bacterial genera remained significantly associated with either trial arm (figure 4).

## Discussion

We successfully nested a large multisite mechanistic study within a large RCT and used a multiomic approach and non-invasive sampling (stool and urine) to explore the impact of supplemental lactoferrin on the microbiome and metabolome. Using this broad, but in-depth approach, we did not demonstrate a significant impact of lactoferrin on the gut microbiome but did identify differences in some unidentifiable metabolomic features. This relative lack of impact of enteral lactoferrin (mean 3% variance) contrasts with other factors which had much larger impacts including infant age (mean 7% variance) and NICU site (mean 30% variance), which explained up to 40% of variance in VOC composition.

Our findings are consistent with the lack of a clinical impact of lactoferrin in both the overarching RCT (ELFIN) and a second similar, large RCT.^27^ However, current meta-analysis of 5425 infants in 12 trials continues to show a reduction in LOS (Risk Ratio 0.8, 95% CI 0.72 to 0.89^28^), although certainty was rated as low. Few other studies have explored potential microbiomic mechanisms in such large nutritional intervention studies. Sherman demonstrated a reduction in Enterobacteriaceae and *Staphylococci* in 10 infants who received recombinant human lactoferrin in comparison to 12 given placebo.^29^ Grzywacz *et al* saw minimal microbiomic or metabolomic impact in 30 infants receiving bovine lactoferrin supplemented with a probiotic in comparison to 29 receiving only probiotics.^30^ However, neither of these studies adjusted their analyses for potential confounding factors in comparison to our extensive in-depth matching and adjustment.

### Strengths and weaknesses

We successfully collected, stored and transported >30 000 samples and used existing high-quality clinical data making this study acceptable, cost-effective and clinically relevant. Successful pragmatic daily longitudinal sampling meant that samples in predetermined time windows were available for many infants, including those who developed serious morbidities. We controlled and adjusted for factors with an established impact on the gut microbiome namely gestation, health (LOS or NEC development), NICU site, postnatal age, MOM receipt and exposure to the intervention. Using a multiomic approach with multiple longitudinal non-invasive samples of urine and stool allowed us to explore fixed and variable exposures, and we conducted analyses blinded to the allocation group.

The 13 NICU sites reflect a diverse geographical spread across the UK as well as a range of clinical practices. Our data emphasise the strong impact of NICU site on microbiomic and metabolomic outcomes, potentially driven by clinical practices including antibiotic and probiotic use as well as feeding practices, and the potential limitations of similar studies restricted to single sites. The biological activity of lactoferrin is affected by multiple factors including iron binding, surface glycans, processing such as pasteurisation, bovine versus human origin,^31^ and variation between commercial products. This highlights some of the challenges faced when determining the efficacy of ‘bionutrients’ in clinical studies.^32^ Finally, due to cost constraints, we opted to use 16S microbiome analysis rather than metagenomics to allow us to study large numbers; however, we acknowledge that this may not identify changes at species or strain level.

### Meaning

Nesting mechanistic work in RCTs increases understanding of pathophysiology and therapeutics, and non-invasive sampling is both successful and acceptable to parents.^33^ Nutritional interventions are complex; nutrients have different kinetics and dynamics compared with drugs, and supplementation with single components of mammalian milk may lack optimal efficacy due to absence of necessary cofactors. For example, the complex of lactoferrin and osteopontin (another mammalian milk protein with ‘bionutrient’ properties) not only resists proteolysis but also results in more effective uptake by gut epithelial cells, and greater proliferation of intestinal proliferation and differentiation than the individual proteins.^17^ Enteral interventions will only be effective after having been tolerated for a period of time and cannot work when infants are not fed. The median age at enrolment to ELFIN was 4 days. Median onset of LOS and NEC in ELFIN are DOL 12 and 17. Thus, only half of the recruited infants had received greater than 8 days of lactoferrin, meaning lactoferrin impact may not be exerted sufficiently early to impact most cases of NEC and LOS. Animal work suggests an established microbiome is key to lactoferrin driven immunomodulation, which may explain the later metabolic impact of lactoferrin.

### Future directions

Careful development of bionutrient interventions, involving basic scientific study and consideration of efficacy in vivo, could better inform the design of future RCTs but remains challenging in high-risk neonatal populations. RCTs provide a unique opportunity for nested mechanistic studies to improve the validity and understanding of clinical outcomes. Individualised analyses may better inform development of personalised treatments. LOS and NEC continue to result in death and serious morbidity and recent RCTs have failed to show clinical benefits.^21 34^ Greater mechanistic understanding will improve the development of nutritional interventions and future trial design.

## Data Availability

Data are available in a public, open access repository. All sequencing data are available at the ENA under study accession PRJEB47702 (https://www.ebi.ac.uk/ena/browser/view/PRJEB47702?show=reads). Metabolomic and VOC data as well as sample metadata will be made available on reasonable request.
