# Supplementary material for: Mechanisms affecting the gut of preterm infants in enteral feeding trials: a nested cohort within a randomised controlled trial of lactoferrin
Source: Arch Dis Child Fetal Neonatal Ed. 2022 Nov 17;108(3):272–9. doi: 10.1136/archdischild-2022-324477 (PMC10176413; doi:10.1136/archdischild-2022-324477)
Supplement: Supplementary data [file fetalneonatal-2022-324477supp001.pdf]

MAGPIE Study Supplementary materials
MAGPIE_Cohort_info.docx

Contents:

OVERALL COHORT INFORMATION
NICU Site coding (table) - Page 2
Samples collected per NICU site (figure) - Page 3

ANALYSIS COHORT INFORMATION
Longitudinal samples received by NICU site (figure & table) - Page 4
Infants recruited to each trial arm (figure) - Page 5
Samples per timepoint (figures) - Page 6
VOC analysis cohort (table) - Page 7
LCMS analysis cohort (table) - Page 7

Young G, *et al. Arch Dis Child Fetal Neonatal Ed* 2023; 108:272–279. doi: 10.1136/archdischild-2022-324477

NICU Site coding table

Participating NICU	***Site code***
Royal Victoria Infirmary, Newcastle	A
City Hospital Sunderland	B
University Hospital of North Tees	C
James Cook University Hospital	D
Bradford Royal Infirmary	E
Leeds General Infirmary	F
Queen’s Medical Centre, Nottingham	G
Nottingham City Hospital	H
Birmingham Women’s Hospital	I
Jessop Wing, Sheffield	J
Leicester Royal Infirmary	K
Queen Alexandra Hospital, Portsmouth	P
Princess Anne Hospital, Southampton	S

Table shows NICU sites participating in MAGPIE study and associated site codes referred to in analysis

Young G, *et al. Arch Dis Child Fetal Neonatal Ed* 2023; 108:272–279. doi: 10.1136/archdischild-2022-324477

Samples collected per NICU site

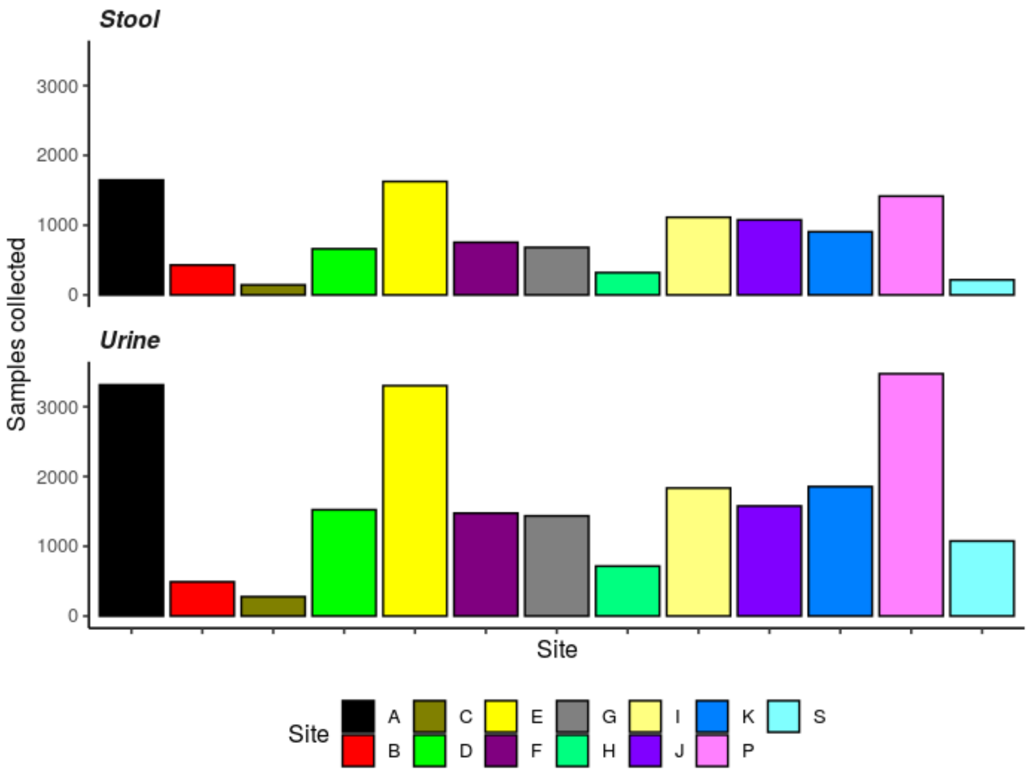

Figure shows total number of stools (upper panel) and urines (lower panel) collected per site during 2 year sampling period of MAGPIE study

Longitudinal samples received by NICU site (figure)

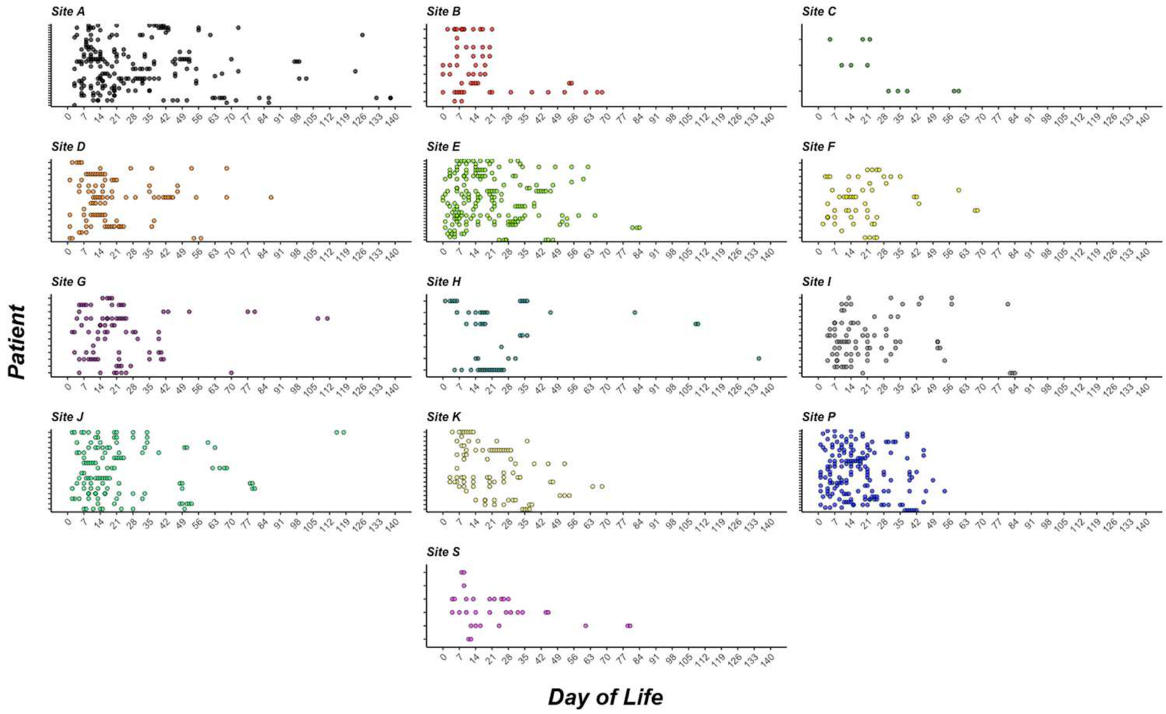

Figure illustrates longitudinal sampling across each of 13 NICU sites involved in MAGPIE recruitment. Each point represents an individual sample selected for analysis. Each panel of the figure represents a different NICU site. Samples are arranged in rows which distinguish samples obtained from one infant or another. Table below numerically outlines the data presented above.

NICU Site	Samples Analysed	Number of Patients
Site A	205	34
Site B	57	9
Site C	11	3
Site D	95	14
Site E	187	27
Site F	56	12
Site G	83	12
Site H	61	7
Site I	80	13
Site J	123	16
Site K	88	18
Site P	171	30
Site S	32	6

Infants recruited to each trial arm

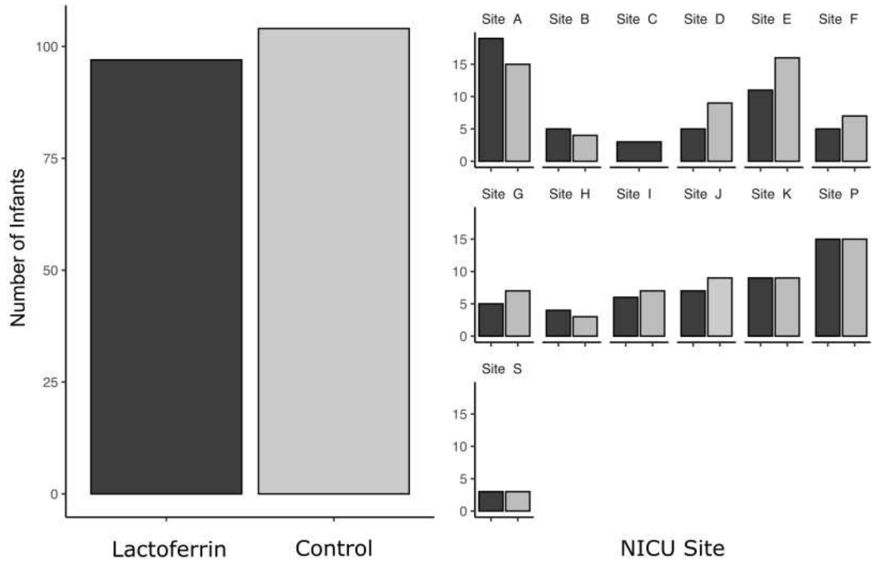

Figure shows number of infants recruited to each trial arm in the MAGPIE analysis cohort overall (left panel) and when stratified by NICU site (right panel).

Young G, *et al. Arch Dis Child Fetal Neonatal Ed* 2023; 108:272–279. doi: 10.1136/archdischild-2022-324477

Samples per timepoint

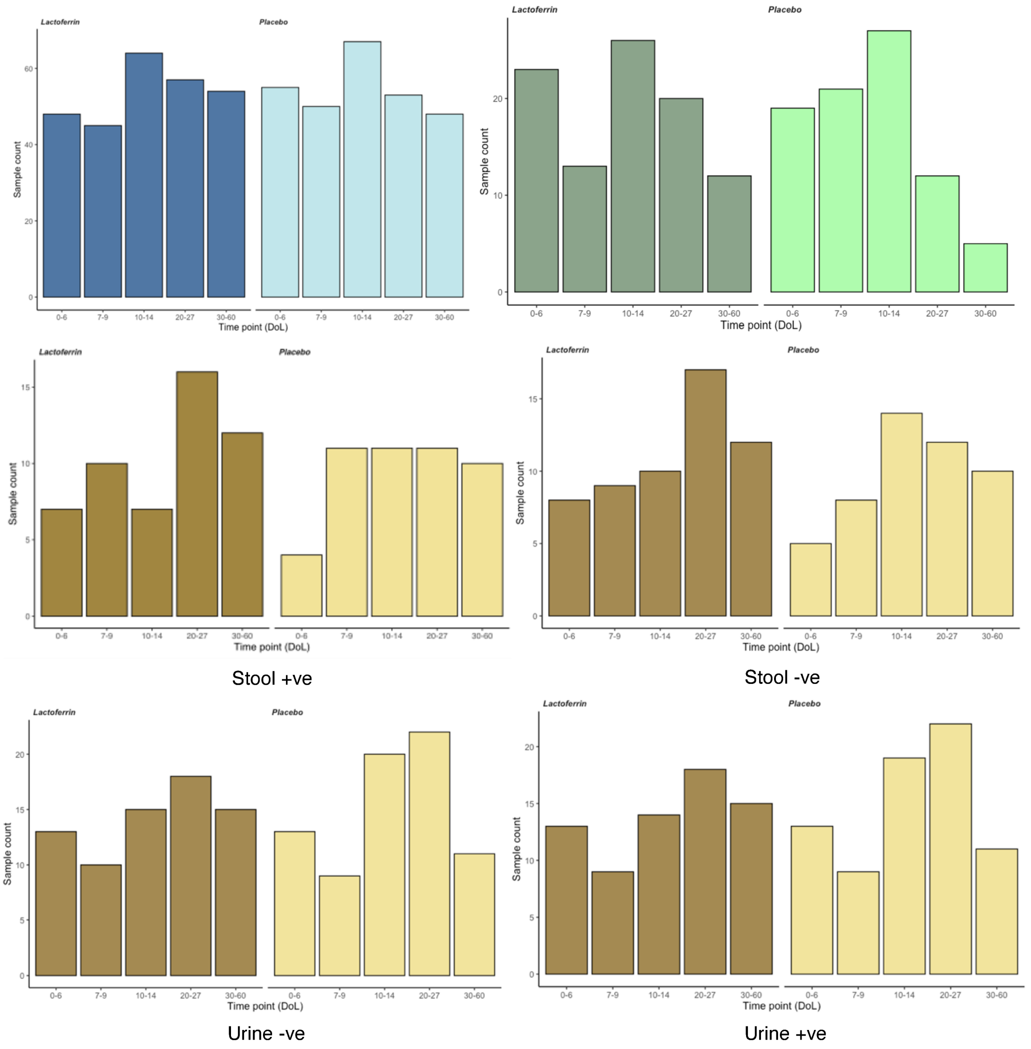

Figure shows number of samples included in MAGPIE analysis cohort at each of 5 pre-determined analysis timepoints. Sample numbers are stratified and shaded by intervention trial arm (dark = lactoferrin; light = placebo) and coloured by analysis mode (blue = microbiota; green = VOC; yellow = metabolites)

BMJ Publishing Group Limited (BMJ) disclaims all liability and responsibility arising from any reliance placed on this supplemental material which has been supplied by the author(s)

VOC Cohort

	VOC		***Stool***
samples *(infants)*	ALL	***Total***	317 *(117)*
		LF	154 (62)
		Ctrl	160 (55)
	No Disease	***Total***	168 *(69)*
		LF	84 (38)
		Ctrl	84 (31)
	Metabolite features (IQR)	***Total***	36
		LF	20 *(14-25)*
		Ctrl	20 *(16 - 24)*

Tables illustrate the number of samples (and infants) included in each trial arm of VOC (above) and LCMS (below) analysis cohorts.

LCMS Cohort

	LCMS		***Stool***		***Urine***	
			pos	*neg*	*pos*	*neg*
samples *(infants)*	ALL	***Total***	170 *(80)*	176 *(81)*	232 *(93)*	236 *(93)*
		LF	82 *(38)*	85 *(39)*	106 *(43)*	109 *(44)*
		Ctrl	88 *(42)*	91 *(42)*	126 *(48)*	127 *(48)*
	No Disease	***Total***	124 *(52)*	125 *(52)*	152 *(53)*	154 *(53)*
		LF	57 *(25)*	57 *(25)*	62 *(26)*	63 *(26)*
		Ctrl	67 *(27)*	68 *(27)*	90 *(27)*	91 *(27)*
	Metabolite features	***Total***	5263	3510	4908	3974
