# Supplementary material for: Mechanisms affecting the gut of preterm infants in enteral feeding trials: a nested cohort within a randomised controlled trial of lactoferrin
Source: Arch Dis Child Fetal Neonatal Ed. 2022 Nov 17;108(3):272–9. doi: 10.1136/archdischild-2022-324477 (PMC10176413; doi:10.1136/archdischild-2022-324477)
Supplement: Supplementary data [file fetalneonatal-2022-324477supp002.pdf]

Metadata collected

Data collected for the ELFIN study was transferred to the MAGPIE team by electronic encrypted data transfer from the clinical trials unit (NPEU). Data provided is outlined below along with description of how this data was managed during analysis *(categorical or continuous)*:

- Hospital site *(categorical)*
- anonymised ELFIN and MAGPIE study number *(categorical)*
- Maternal age, use of antenatal steroids, absence/reversed end-diastolic umbilical arterial blood flow, duration of membrane rupture *(not included in analysis)*
- Delivery mode *(categorical)*
- Gestational age *(continuous)*
- Birthweight (g) *(continuous)*
- Twin/triplet status (multiple birth) *(categorical)*
- Daily use of parenteral nutrition *(categorical)*
- Milk type received on each postnatal day (either breast, formula or mixed) *(categorical)*
- Receipt of ELFIN IMP, and ELFIN trial group (Blinding was maintained until analysis was complete). *(categorical)*

Supplemental data collected from bedside samples logs as part of the MAGPIE study included:

- Date of sample collection *(continuous)*
- Daily information on antibiotic type and use of prophylactic antifungal agents (either nystatin or fluconazole) *(not included in analysis)*
- Use of probiotics (all NICUs who used probiotics (A, D & E) used the same commercially available product which includes 3 species – *B infantis, B bifidum, and L acidophilus*. *(categorical)*
- Unique sample code *(categorical)*
- Shipment date (transfer to central laboratory) *(not included in analysis)*

Sample collection and transport

Stool and urine collection pots, spoons and vials were provided by the research team and were the same for each NICU. Stool samples were collected at routine nursing care times from the nappy using a clean disposable plastic spoon and placed in a glass pot with a lid. Urine samples were collected according to standard NICU procedure which involved collection of urine passed spontaneously onto a sterile cotton wool ball, then squeezing this out using a sterile syringe and aliquoting it into two 2 mL cryovials. Samples were labelled and placed in a -20 ºC freezer located on the NICU as soon as possible. Samples were transferred frozen, in batches of approximately 500 samples, from the local NICU to Northumbria University then stored at -80 ºC prior to analyses. Courier transport of samples occurred every 2-4 months.

Sample pulling & splitting

Stool samples were removed from -80 °C in randomised batches of 188 samples and stored on ice. In turn, each sample was transferred to a class II laminar flow hood and split in to N x 0.1 g aliquots using a sterile scalpel (where N = number of analyses required). Aliquots for GCMS or LCMS were transferred to a sterile glass pot and immediately sealed along with a sample-splitting negative control, consisting of an empty sterile glass pot sealed at the same time as the samples. Aliquot for 16S rRNA analysis of microbial communities were transferred to an individual well of DNeasy PowerSoil HTP 96 Kit (QIAGEN, DE) bead-beating plates and stored frozen at -80 °C overnight prior to isolation of bacterial DNA.

Isolation of genomic DNA from stools

Stool samples for bacterial community analysis were defrosted at ambient temperature in individual wells of a DNeasy PowerSoil HTP 96 Kit bead-beating plate. Extraction was performed as per manufacturer’s instructions, utilising 2 x 10-minute bead beating steps at 20 Hz on a TissueLyser II (QIAGEN, DE) with minor adjustments. Centrifugation speeds were lowered to 2250 x g and times extended due to limitations of laboratory equipment. 15-minute incubation at ambient temperature following addition of elution buffer, was employed to maximise yields of DNA eluted from silica filters.

DNA isolation was performed in batches of 188 samples across two HTP 96 well plates. Each plate contained an extraction kit negative (an empty well processed as per the kit extraction protocol), and one well which remained completely blank for inclusion of sequencing negative during library preparation protocol. Isolated DNA was and stored at -80 °C until sequencing library preparation.

Bacterial 16S rRNA gene sequencing library preparation

Sequencing libraries were prepared via the protocol described by Kozich et al., using V4 region primers (515f – 5' GTG CCA GCM GCC GCG GTA A 3'; 806r - 5' GGA CTA CHV GGG TWT CTA AT 3'). Amplicons were normalised and cleaned with the SequalPrep normalisation kit (Thermo Fisher, MA) before pooling barcoded amplicons in multiplex libraries containing 96 samples. Each sequencing library contained a kit negative and sequencing negative and was sequenced on the Illumina MiSeq platform (Illumina, CA), using V2 2 x 250 chemistry.

Sequence data processing and microbial community analysis

Paired end sequences were merged, quality filtered, clustered in to OTUs and assigned consensus taxonomies based on sequence similarity to the SILVA 16S rRNA gene database using Mothur v.1.42.363. OTUs were merged at the genus level for analysis unless otherwise stated. OTUs contributing fewer than 10 reads across all samples were culled from analysis. Libraries were rarefied to 1000 reads for compositional analysis, retaining 94% of samples. All sequencing data is available at the ENA under study accession PRJEB47702 (https://www.ebi.ac.uk/ena/browser/view/PRJEB47702?show=reads).

Preparation of urines for UHPLC-MS analysis

The specific gravity of each urine sample was measured using a Reichert TS 400 Total Solids Refractometer (Fisher Scientific). Urine samples were thawed on ice, vortexed (10 s), then centrifuged (4°C, 5,000-g, 5 min). 100 µL of supernatant was loaded onto the refractometer and the urine specific gravity (USG) measurement was taken. A USG linear standard curve was created by taking the urine sample with the highest USG and carrying out a serial dilution

of the urine with water (neat, 80%, 60%, 40%, 20%, 10%, 5%, blank [LC-MS grade water, VWR]). The equation of the line from the standard curve was used to calculate the dilution factor of each urine sample based on their USG measurement. Each sample was diluted with water (LC-MS grade, VWR) to make samples equivalent in concentration according to the USG reading. 50 µL of the diluted urine sample was mixed with 150 µL ice-cold 50:50 methanol:water (LC-MS grade, VWR). Samples were vortex mixed (15 seconds), centrifuged (13,000×g, 20 min, 4°C) and 100 µL of the clear supernatant was transferred to a glass LC autosampler vial (VI-04-12-02RVG 300µL Plastic, Chromatography Direct, UK) for immediate UHPLC-MS analysis. A single pooled QC sample was prepared by combining 25 µL aliquots from each diluted urine sample and vortex mixing (2 minutes). Aliquots (50 µL) of the pooled QC sample were prepared as defined above for the urine samples. Extraction blank samples were prepared by adding 100 µL water to a blank urine collection tube and vortexing (1 minute). 50 µL of this solution was removed and prepared as defined above for the samples.

Preparation of stools for UHPLC-MS analysis

Frozen feces samples were weighed (mass range 50-124 mg). 80:20 methanol:water (LC-MS grade, VWR) was added to each sample – the volume of added solvent was normalised to feces mass (15 µL of solvent was added per mg of mass). Samples were homogenised in a bead-based homogeniser (Precellys24 system and 2 mL tubes containing CK14 ceramic beads, Stretton Scientific) at 6400rpm for 2 bursts of 10 seconds separated by a 10 second break. The homogenate was transferred to an Eppendorf and centrifuged (13,000×g, 20 minutes, 4°C). 650 µL of supernatant (equivalent to 43 mg of biomass) was removed to a clean tube and dried in a SpeedVac concentrator (Thermo Fisher). To create QC samples, an additional 100 µL of supernatant was taken from each sample, pooled, vortex mixed (2 min), aliquoted into 650 µL aliquots and then dried via SpeedVac. Dried extracts were re-suspended in 430 µL 1.5:2.5 methanol:water, vortexed (30 s) and centrifuged (13,000×g, 20 min, 4°C). 100 µL of the clear supernatant was transferred to a glass LC auto-sampler vial (VI-04-12-

02RVG 300µL Plastic, Chromatography Direct, UK) for immediate UHPLC-MS analysis. Several extraction blanks were prepared. 50-100 mg pieces of nappies, sampling equipment (plastic spoons) or 100 µL water were extracted in the same way as for the samples.

Ultra-High Performance Liquid Chromatography-Mass Spectrometry

The samples were maintained at 4°C and analysed applying an UHPLC-MS method using a Dionex UltiMate 3000 Rapid Separation LC system (Thermo Fisher Scientific, MA, USA) coupled with a heated electrospray Q Exactive Focus mass spectrometer (Thermo Fisher Scientific, MA, USA). Polar extracts were analysed on a Hypersil GOLD C18-aqueous column (100 x 2.1 mm, 2.6µm, Thermo Fisher Scientific, MA, USA). Mobile phase A consisted of water with 0.1% formic acid (LC-MS grade, Sigma) and mobile phase B consisted methanol with 0.1% formic acid. For both positive and negative ion mode the initial flow rate was set for 0.30 mL.min-1 rising to 0.4 min-1 between 11-14.9 min. The gradient was as follows: t=0.0, 1% B; t=0.5, 1% B; t=2.0, 50% B; t=10.5, 99% B; t=11.0, 99% B; t=11.5, 1% B; t=15, 1% B; all changes were linear with curve = 5 except: 2 - 10.5 min (curve=8) and 11.5 - 15 min (curve=7). The column temperature was set to 45 °C and the injection volume was 2 µL. Data were acquired in positive and negative ionisation modes separately within the mass range of 100 – 1500 m/z at resolution 70,000 (FWHM at m/z 200). Ion source parameters were set as follows: Sheath gas = 30 arbitrary units, Aux gas = 13 arbitrary units, Sweep gas = 0 arbitrary units, Spray Voltage = 3.2kV (positive ion) / 2.7kV (negative ion), Capillary temp. = 350 °C, Aux gas heater temp. = 400 °C. Data dependent MS2 in 'Discovery mode' was used for the MS/MS spectra acquisition using following settings: resolution = 17,500 (FWHM at m/z 200); Isolation width = 3.0 m/z; stepped collision energies (stepped CE) = 20, 40, 100 [positive ion mode] / 40, 60, 130 [negative ion mode]. MS/MS mass spectra were acquired in five different precursor m/z ranges: 100 – 260 m/z; 250 – 360 m/z; 350 – 510 m/z; 500 – 700 m/z; 690 – 1500 m/z. A Thermo ExactiveTune 2.8 SP1 build 2806 was used as instrument control software in both cases and data were acquired in profile mode. Quality control (QC) samples were analysed

as the first ten injections and then every seventh injection with two QC samples at the end of the analytical batch. Two blank samples were analysed, the first as the 6th injection and then the second at the end of each batch.

Mass spectrometry raw data processing

Raw data acquired in each analytical batch were converted from the instrument-specific format to the mzML file format applying the open access ProteoWizard (version 3.0.11417) msconvert too.77 During this procedure, peak picking and centroiding, were achieved using vendor algorithms. Isotopologue Parameter Optimization (IPO - version 1.0.0, using XCMS - version 1.46.0)79 was used to perform automatic optimization of XCMS80 peak picking parameters. For centWave peak picking algorithm following parameters and ranges were used: min_peakwidth (from 2 to 10); max_peakwidth (from 20 to 60); ppm (from 5 to 15); mzdiff (-0.001 to 0.01); snthresh (10); noise (10000); prefilter (3); value_of_prefilter (100); mzCenterFun (wMean); integrate (1); fitgauss (FALSE); verbose.columns (FALSE). Optimised XCMS parameters for raw data files deconvolution were applied and a data matrix of metabolite features (m/z-retention time pairs) versus samples was constructed with peak areas provided where the metabolite feature was detected for each sample.

Peak matrix processing

The data for pooled QC samples were applied to perform QC filtering. The first five QCs for each batch were used to equilibrate the analytical system and therefore subsequently removed from the data before the data was processed and analysed. The data from the pooled QC samples were used to apply QC filtering. For each metabolite feature detected QC samples 1-8 were removed (i.e. leaving a blank and 2 QCs at the start of each batch) and the relative standard deviation and percentage detection rate were calculated using the remaining QC samples. Blank samples at the start and end of a run were used to remove features from non-biological origins. Any feature with an average QC intensity less than 20 times the

average intensity of the blanks was removed. Any samples with >50% missing values were excluded from further analysis. Metabolite features with an RSD > 30% and present in less than 90% of the QC samples were deleted from the dataset. Features with a <50% detection rate over all samples were also removed.

Metabolite Annotation

Putative annotation of metabolites or metabolite groups was performed by applying the BEAMS workflows, which is maintained by Phenome Centre Birmingham and available on GitHub (https://github.com/computational-metabolomics/beams). We applied 5 ppm mass error and a retention time range of 5 s in feature grouping and molecular formula and metabolite matching. As different metabolites can be detected with the same accurate m/z (for example, isomers with the same molecular formula), multiple annotations could be observed for a single detected metabolite feature. Also, a single metabolite could be detected as multiple molecules, particularly as a different type of ion (e.g., protonated and sodiated ions). All molecules were annotated according to guidelines for reporting of chemical analysis results, specifically to Metabolomics Standards Initiative level 2 (2007).

GCMS analyses of stool

VOC analysis of stools occurred in two batches of 36 and 116 samples. Analysis was performed using gas-chromatography mass- spectrometry (GC-MS) on a PerkinElmer Clarus 500 GC-MS quadrupole benchtop system (Beaconsfield, UK) and Combi PAL auto-sampler (CTC Analytics, Switzerland). VOCs were extracted using solid phase micro-extraction with a divinylbenzene-carboxen- polydimethylsiloxane (DVB-CAR-PDMS, DVB-CAR-PDMS (Sigma-Aldrich, Dorset, UK)) coated fibre, otherwise the protocol and GC-MS conditions were the same as published by Reade *et al*. The fibre was pre-conditioned before use, in accordance with the manufacturer's manual. Samples were heated to 60°C for 30 mins at prior to fibre

exposure. The fibre was exposed to the headspace gases at 60 °C for 20 mins, then VOCs were thermally desorbed for 5 mins at 220 °C.

The GC column used was a 60m Zebron ZB-624 (inner diameter 0.25 mm, length 60 m, film thickness 1.4 µm (Phenomenex, Macclesfield, UK)). The carrier gas was 99.996% pure helium (BOC, Sheffield, UK) which was passed through a helium purification system, ExcelasorbTM (Supelco) at 1 mL/min. The initial temperature of the GC oven was set at 40°C and held for 2 minutes before increasing to 220°C at a rate of 5°C/min and held for 4 mins with a total run time of 41 mins. The MS was operated in electron impact ionization EI+ mode, scanning from ion mass fragments 10 to 300 m/z with an interscan delay of 0.1 sec and a resolution of 1000 at FWHM (Full Width at Half Maximum). The sensitivity of the instrument was determined with 2-pentanone only and varies for other compounds. The limit of detection was 3 times the signal/noise ratio, of the method for 2-pentanone with DVB-CAR-PDMS is 16 ppm.

VOC data and statistical Analysis

VOC data were processed using Automated Mass Spectral Deconvolution System (AMDIS-version 2.71, 2012) coupled to the National Institute of Standards and Technology (NIST) mass spectral library (version 2.3, 2017) to putatively identify VOCs. The R package Metab was used to align data. VOCs data were analysed with R (version 3.4.2) R Core Team.74 Firstly, the VOCs table was adjusted as follow: only compounds observed in at least 25% of samples were kept, normalization by natural log transformation was performed using the log1p() function and missing values were adjusted to 1.

Feature normalisation methods

Bacterial features (16s rRNA gene sequencing)	• Culled features <10 reads across all samples<br>• Rarefied samples to 1000 reads

VOC features (GCMS)	• Culled features present in <25% samples<br>• Missing values imputed<br>• Features nlog transformed per sample
Metabolite features (UHPLC-MS)	• Culled features with average intensity <20 x intensity in blanks<br>• Culled features present in <50% samples<br>• Features PQN normalised against blanks<br>• Missing values imputed<br>• Features glog transformed per sample

Assessing collinearity of variables in GLMMs

Collinearity of variables is a confounding factor when constructing linear mixed models to assess the relationships between mixed categorical and continuous variables and count response data. To assess collinearity of variables we employed Variance Inflation Factors (VIFs) using the '*car::vif*' function in R. Any variables with VIFs greater than a pre-defined threshold (2.00) were classified as potentially collinear. Where collinear variables were identified as not significantly associated with the response data the collinear variables were systematically removed from the models in descending order (starting with the highest VIF score), until all VIF scores were below the pre-defined threshold. Where variable collinearity impacted the significance of the relationship between variables and response data this was highlighted in the results (*Table: Corrected alpha diversity comparisons per individual analysis mode, Page 5 – Supplementary results*)
