# Supplementary material for: Mechanisms affecting the gut of preterm infants in enteral feeding trials: a nested cohort within a randomised controlled trial of lactoferrin
Source: Arch Dis Child Fetal Neonatal Ed. 2022 Nov 17;108(3):272–9. doi: 10.1136/archdischild-2022-324477 (PMC10176413; doi:10.1136/archdischild-2022-324477)
Supplement: Supplementary data [file fetalneonatal-2022-324477supp003.pdf]

MAGPIE study supplementary materials
Supplementary results

*Contents:*


| | | |
|---|---|---|
| *16S analysis library sizes (figure)* | - | *Page 2* |
| *Pairwise Wilcoxon test 16S analysis library size between samples & controls (table)* | - | *Page 2* |
| *Boxplot 16S analysis library size between samples & controls (figure)* | - | *Page 2* |
| *Overall bacterial composition over time (figure)* | - | *Page 3* |
| *Prevalence of VOC features identified (table)* | - | *Page 4* |
| *Corrected alpha diversity comparisons per individual analysis mode (table)* | - | *Page 5* |
| *Corrected beta diversity comparisons per individual analysis mode (table)* | - | *Page 6* |
| *Kruskal-wallis test: Concordant Vs Discordant siblings average sample composition dissimilarity (table)* | - | *Page 7* |
| *Sitewise MaAsLin results: Significantly discriminant features between lactoferrin and placebo samples in each analysis mode* | - | *Page 7* |
| *Merging of positive and negative LCMS ion modes does not impact results (table)* | - | *Page 8* |




Young G, *et al. Arch Dis Child Fetal Neonatal Ed* 2023; 108:272–279. doi: 10.1136/archdischild-2022-324477

*16S analysis library size*

A median of 38,677 reads (IQR 22,206-63,040) shared across 874 genera were sequenced in the 1304 samples undergoing bacterial community analysis.

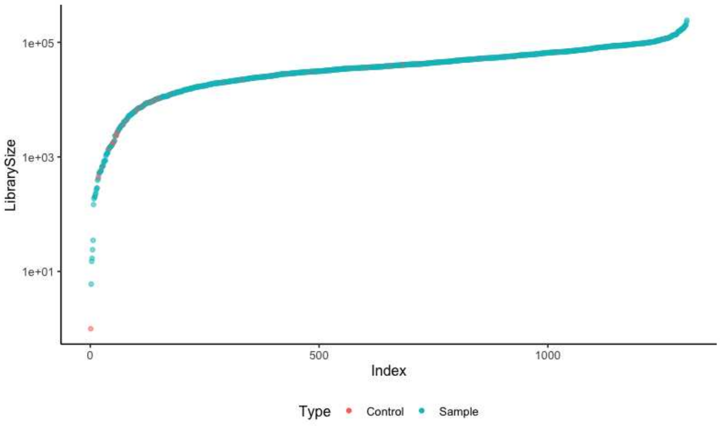


*Figure illustrates raw library sizes of samples (blue) and controls (red) prior to rarefaction and analysis. Each point represents an individual sample. Library size is expressed on a log scale (y axis) with libraries arranged in increasing size order along the x axis*

*Pairwise comparison 16S analysis read depth between samples and controls*

| | Seq – control | Kit – control | Samples |
|---|---|---|---|
| **Kit – control** | 1 | - | - |
| **Samples** | < 0.0001 | 0.005 | - |
| **Seq + control** | 0.00031 | 0.003 | 0.48 |

*Table shows results of pairwise Wilcoxon test comparing mean library size of samples and controls performed in the 16S rRNA sequencing analysis of the MAGPIE analysis cohort. P values are corrected for multiple hypothesis testing using the method of Bonferroni. This comparison is visualized in the figure below where boxes are coloured by sample (dark) or control (light) status. Limits of the boxes extend to the 1st and 3rd quartiles. The centre line marks the median. Whiskers extend to the full range of the data, excluding outliers.*

*Boxplot 16S analysis library size between samples and controls*
*Samples contained significantly more reads than controls (KW test; P < 0.0001)*

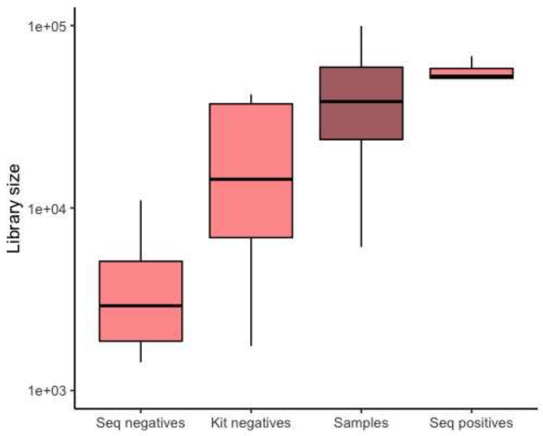


Control compositions were also significantly different to samples (ANOSIM[999 perms] R2 = 0.14, P = 0.002)

*Overall sample compositions over time*

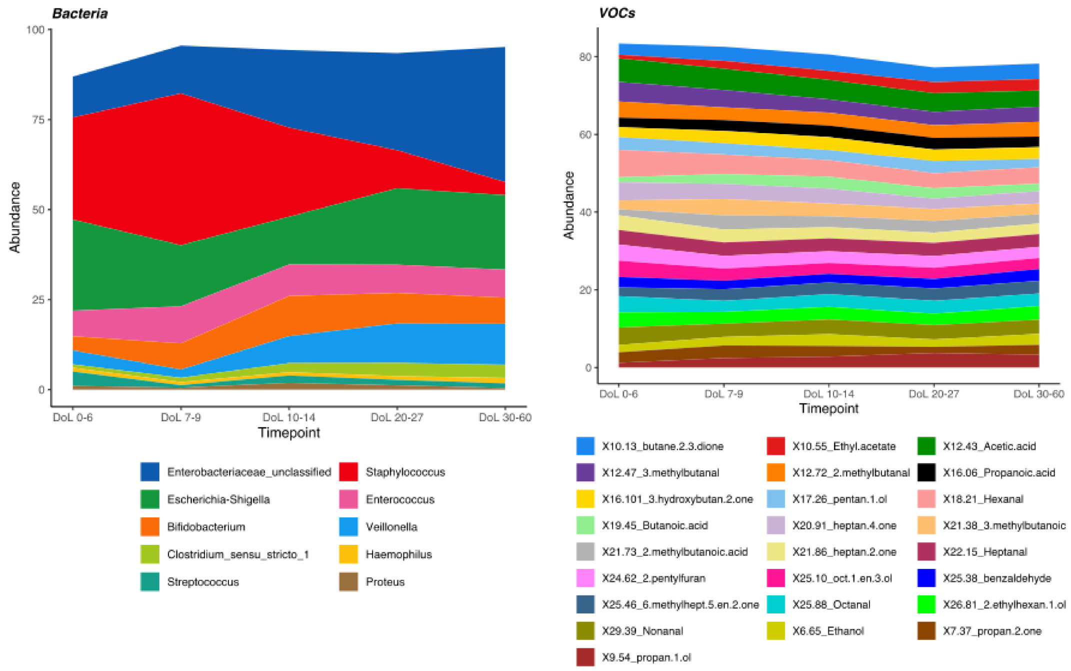


*Figures illustrate the overall bacterial (left panel) and VOC (right panel) compositions of samples analysed in the MAGPIE study. Average abundance of features were calculated by timepoint (with one sample provided per patient, per timepoint) to illustrate to progression of community compositions over time. The top 10 most abundant features, accounting for >90% total sequence reads) are plotted on the left panel. The top 25 most abundant features (accounting for >80% total feature abundance) are plotted on the right panel.*



Young G, *et al. Arch Dis Child Fetal Neonatal Ed* 2023; 108:272–279. doi: 10.1136/archdischild-2022-324477

*Prevalence of VOC features identified*

| VOC Feature | Count | Prevalence (%) |
|---|---|---|
| **Hexanal** | 289 | 91.17 |
| **Acetic-acid** | 283 | 89.27 |
| **Nonanal** | 263 | 82.97 |
| **3-methylbutanal** | 253 | 79.81 |
| **Heptanal** | 245 | 77.29 |
| **Heptan-4-one** | 232 | 73.19 |
| **Butane-2-3-dione** | 231 | 72.87 |
| **Octanal** | 228 | 71.92 |
| **2-Methylbutanal** | 219 | 69.09 |
| **2-Pentylfuran** | 218 | 68.77 |
| **Oct-1-en-3-ol** | 200 | 63.09 |
| **3-Methylbutanoic-acid** | 194 | 61.20 |
| **2-Ethylhexan-1-ol** | 193 | 60.88 |
| **6-Methylhept-5-en-2-one** | 192 | 60.57 |
| **Heptan-2-one** | 191 | 60.25 |
| **3-Hydroxybutan-2-one** | 171 | 53.94 |
| **Propan-1-ol** | 168 | 53.00 |
| **Propanoic-acid** | 167 | 52.68 |
| **2-Methylbutanoic-acid** | 166 | 52.37 |
| **Benzaldehyde** | 166 | 52.37 |
| **Pentan-1-ol** | 162 | 51.10 |
| **Propan-2-one** | 148 | 46.69 |
| **2-Phenylacetaldehyde** | 140 | 44.16 |
| **Butanoic-acid** | 139 | 43.85 |
| **Ethyl-acetate** | 127 | 40.06 |
| **2-Methylpropanal** | 124 | 39.12 |
| **Ethanol** | 123 | 38.80 |
| **Propyl-acetate** | 122 | 38.49 |
| **Methyl--Z--N-hydroxybenzenecarboximidate** | 122 | 38.49 |
| **Propyl-propanoate** | 115 | 36.28 |
| **Ethyl-propanoate** | 112 | 35.33 |
| **4R-1-methyl-4-prop-1-en-2-ylcyclohexene** | 105 | 33.12 |
| **Hexan-1-ol** | 104 | 32.81 |
| **Ethylbenzene** | 92 | 29.02 |
| **1-4-Xylene** | 89 | 28.08 |
| **2-Ethyl-Furan** | 85 | 26.81 |

*Table highlights prevalence of 36 VOC features present >25% samples across MAGPIE analysis cohort.*

*Corrected alpha diversity comparisons per individual analysis mode*

| ***Bacterial diversity (Shannon)*** | *Timepoint* | *Lactoferrin* | *NICU Site* | *Feed type* | *Health status* | *Gestational age* | *Birthweight* |
|---|---|---|---|---|---|---|---|
| *Chisq* | 49.7165 | 0.3916 | 11.3684 | 1.3312 | 0.3398 | 0.0087 | 5.273 |
| *Df* | 4 | 1 | 12 | 2 | 1 | 1 | 1 |
| *Pr(>Chisq)* | ***4.14E-10*** | 0.53147 | 0.49764 | 0.51397 | 0.55997 | 0.92565 | ***0.02166**** |
| *VIF* | 1.01 | 1.02 | 1.02 | 1.07 | 1.14 | 2.06 | 2.08 |

| ***Bacterial richness*** | *Timepoint* | *Lactoferrin* | *NICU Site* | *Feed type* | *Health status* | *Gestational age* | *Birthweight* |
|---|---|---|---|---|---|---|---|
| *Chisq* | 23.9593 | 0.9707 | 11.8143 | 2.1751 | 1.7163 | 0.0636 | 1.4256 |
| *Df* | 4 | 1 | 12 | 2 | 1 | 1 | 1 |
| *Pr(>Chisq)* | ***8.14E-5*** | 0.3245 | 0.4607 | 0.3370 | 0.1902 | 0.8009 | 0.2325 |
| *VIF* | 1.02 | 1.02 | 1.03 | 1.08 | 1.15 | 2.09 | 2.10 |

| ***VOCs*** | *Timepoint* | *Lactoferrin* | *NICU Site* | *Feed type* | *Health status* | *Gestational age* | *Birthweight* |
|---|---|---|---|---|---|---|---|
| *Chisq* | 88.6248 | 0.0037 | 19.4176 | 1.8814 | 2.5843 | 1.4503 | 1.6471 |
| *Df* | 4 | 1 | 11 | 2 | 1 | N/A | N/A |
| *Pr(>Chisq)* | ***<2e-16*** | 0.9514 | 0.054 | 0.3903 | 0.1079 | 0.2285 | 0.1993 |
| *VIF* | 1.08 | 1.10 | 1.07 | 1.16 | 1.27 | 2.36 | 2.39 |

| ***Metbolites (s)*** | *Timepoint* | *Lactoferrin* | *NICU Site* | *Feed type* | *Health status* | *Gestational age* | *Birthweight* |
|---|---|---|---|---|---|---|---|
| *Chisq* | 22.1221 | 2.276 | 51.5225 | 6.5257 | 5.1053 | 0.6661 | 0.4842 |
| *Df* | 4 | 1 | 12 | 3 | 1 | 1 | 1 |
| *Pr(>Chisq)* | ***1.90E-04*** | 0.1313886 | ***7.532E-07*** | 0.0886546 | ***0.0238534*** | 0.4144098 | 0.4865356 |
| *VIF* | 1.10 | 1.11 | 1.08 | 1.24 | 1.31 | 2.58 | 2.51 |

| ***Metabolites (u)*** | *Timepoint* | *Lactoferrin* | *NICU Site* | *Feed type* | *Health status* | *Gestational age* | *Birthweight* |
|---|---|---|---|---|---|---|---|
| *Chisq* | 10.9917 | 0.4689 | 11.1489 | 4.8315 | 0.0734 | 0.4215 | 1.7629 |
| *Df* | 4.00E+00 | 1 | 11 | 3 | 1 | 1 | 1 |
| *Pr(>Chisq)* | ***0.02666*** | 0.49348 | 0.43087 | 0.18456 | 0.78642 | 0.5162 | 0.18427 |
| *VIF* | 1.09 | 1.08 | 1.08 | 1.16 | 1.31 | 2.81 | 2.60 |

*Table presents significant associations between each clinical variable recorded and alpha diversity measures across each analysis mode utilized per sample. Significant relationships are highlighted in bold & colour according to the analysis mode used. Collinear variables were identified using VIF scores as explained in Supplementary methods. Collinear variables with significant relationships are depicted with an asterisk.*

*Corrected beta diversity comparisons per individual analysis mode*

**Beta-diversity**

**Bacteria** *(Bray-Curtis dissimilarity)*

| DOL | Variable | df | SumOfSqs | R2 | F | Pr(>F) | |
|---|---|---|---|---|---|---|---|
| DOL 0-6 | Lactoferrin | 1 | 0.248 | 0.00772 | 0.8795 | 0.458 | |
| | NICU site | 12 | 4.289 | 0.13353 | 1.2678 | 0.099 | . |
| | Health status | 1 | 0.284 | 0.00883 | 1.006 | 0.386 | |
| | Feed type | 2 | 1.132 | 0.03525 | 2.0081 | 0.026 | * |
| | Gestational age | 1 | 0.3 | 0.00935 | 1.0657 | 0.343 | |
| | Birthweight | 1 | 0.351 | 0.01094 | 1.2464 | 0.236 | |
| | Trial exposure days | 1 | 1.165 | 0.03627 | 4.1325 | 0.002 | ** |
| | Residuals | 83 | 23.4 | 0.72847 | | | |
| | Total | 102 | 32.122 | 1 | | | |
| DOL 7-9 | Lactoferrin | 1 | 0.2642 | 0.00985 | 1.1346 | 0.328 | |
| | NICU site | 10 | 6.0253 | 0.22461 | 2.5877 | 0.001 | *** |
| | Health status | 1 | 0.39 | 0.01454 | 1.6749 | 0.154 | |
| | Feed type | 2 | 0.9433 | 0.03516 | 2.0255 | 0.052 | . |
| | Gestational age | 1 | 0.1425 | 0.00531 | 0.612 | 0.687 | |
| | Birthweight | 1 | 0.3494 | 0.01302 | 1.5004 | 0.217 | |
| | Trial exposure days | 1 | 0.1767 | 0.00659 | 0.7588 | 0.54 | |
| | Residuals | 77 | 17.9293 | 0.66835 | | | |
| | Total | 94 | 26.8262 | 1 | | | |
| DOL 10-14 | Lactoferrin | 1 | 0.199 | 0.00475 | 0.6939 | 0.652 | |
| | NICU site | 12 | 5.858 | 0.1403 | 1.7065 | 0.001 | *** |
| | Health status | 1 | 0.169 | 0.00406 | 0.5924 | 0.779 | |
| | Feed type | 2 | 0.843 | 0.02018 | 1.4725 | 0.12 | |
| | Gestational age | 1 | 0.106 | 0.00254 | 0.3711 | 0.908 | |
| | Birthweight | 1 | 0.363 | 0.0087 | 1.2695 | 0.259 | |
| | Trial exposure days | 1 | 0.358 | 0.00858 | 1.2517 | 0.26 | |
| | Residuals | 111 | 31.755 | 0.76051 | | | |
| | Total | 130 | 41.755 | 1 | | | |
| DOL 20-27 | Lactoferrin | 1 | 0.399 | 0.01196 | 1.4753 | 0.177 | |
| | NICU site | 12 | 5.118 | 0.15336 | 1.5764 | 0.005 | ** |
| | Health status | 1 | 0.284 | 0.00852 | 1.0503 | 0.381 | |
| | Feed type | 2 | 0.483 | 0.01446 | 0.8921 | 0.538 | |
| | Gestational age | 1 | 0.922 | 0.02763 | 3.4078 | 0.004 | ** |
| | Birthweight | 1 | 1.092 | 0.03272 | 4.0366 | 0.005 | ** |
| | Trial exposure days | 1 | 0.131 | 0.00393 | 0.4846 | 0.811 | |
| | Residuals | 90 | 24.348 | 0.72963 | | | |
| | Total | 109 | 33.37 | 1 | | | |
| DOL 30-60 | Lactoferrin | 1 | 0.1998 | 0.00656 | 0.8572 | 0.47 | |
| | NICU site | 11 | 4.5839 | 0.15048 | 1.7879 | 0.001 | *** |
| | Health status | 1 | 0.5959 | 0.01956 | 2.5567 | 0.031 | * |
| | Feed type | 2 | 0.5638 | 0.01851 | 1.2095 | 0.282 | |
| | Gestational age | 1 | 0.3778 | 0.0124 | 1.6211 | 0.16 | |
| | Birthweight | 1 | 0.1809 | 0.00594 | 0.7761 | 0.554 | |
| | Trial exposure days | 1 | 0.4598 | 0.0151 | 1.9729 | 0.077 | . |
| | Residuals | 96 | 22.3746 | 0.73453 | | | |
| | Total | 114 | 30.461 | 1 | | | |

**VOCs** *(Canberra dissimilarity)*

| DOL | Variable | df | SumOfSqs | R2 | F | Pr(>F) | |
|---|---|---|---|---|---|---|---|
| DOL 0-6 | Lactoferrin | 1 | 0.0472 | 0.01199 | 0.6256 | 0.737 | |
| | NICU site | 11 | 1.3989 | 0.35517 | 1.6849 | 0.005 | ** |
| | Health status | 1 | 0.3049 | 0.0774 | 4.0392 | 0.002 | ** |
| | Feed type | 2 | 0.1994 | 0.05062 | 1.3209 | 0.201 | |
| | Gestational age | 1 | 0.1875 | 0.0476 | 2.484 | 0.023 | * |
| | Birthweight | 1 | 0.0373 | 0.00946 | 0.4938 | 0.812 | |
| | Trial exposure days | 1 | 0.1889 | 0.04797 | 2.5033 | 0.017 | * |
| | Residuals | 23 | 1.736 | 0.44076 | | | |
| | Total | 41 | 3.9386 | 1 | | | |
| DOL 7-9 | Lactoferrin | 1 | 0.1718 | 0.05015 | 1.5827 | 0.17 | |
| | NICU site | 8 | 1.1792 | 0.34428 | 1.3582 | 0.126 | |
| | Health status | 1 | 0.288 | 0.0841 | 2.6541 | 0.031 | * |
| | Feed type | 2 | 0.1627 | 0.04752 | 0.7498 | 0.669 | |
| | Gestational age | 1 | 0.0545 | 0.01591 | 0.5022 | 0.8 | |
| | Birthweight | 1 | 0.0821 | 0.02396 | 0.7561 | 0.608 | |
| | Trial exposure days | 1 | 0.2168 | 0.06329 | 1.9976 | 0.092 | . |
| | Residuals | 15 | 1.6279 | 0.47527 | | | |
| | Total | 30 | 3.4251 | 1 | | | |
| DOL 10-14 | Lactoferrin | 1 | 0.1375 | 0.02806 | 1.5394 | 0.168 | |
| | NICU site | 11 | 1.2914 | 0.26347 | 1.3139 | 0.095 | . |
| | Health status | 1 | 0.0434 | 0.00886 | 0.486 | 0.829 | |
| | Feed type | 2 | 0.1317 | 0.02686 | 0.7367 | 0.73 | |
| | Gestational age | 1 | 0.0804 | 0.0164 | 0.8995 | 0.496 | |
| | Birthweight | 1 | 0.0341 | 0.00696 | 0.3819 | 0.876 | |
| | Trial exposure days | 1 | 0.2282 | 0.04655 | 2.5538 | 0.027 | * |
| | Residuals | 32 | 2.8593 | 0.58335 | | | |
| | Total | 50 | 4.9015 | 1 | | | |
| DOL 20-27 | Lactoferrin | 1 | 0.09781 | 0.04463 | 1.4327 | 0.203 | |
| | NICU site | 10 | 0.72191 | 0.32941 | 1.0574 | 0.435 | |
| | Health status | 1 | 0.02699 | 0.01231 | 0.3953 | 0.883 | |
| | Feed type | 2 | 0.08486 | 0.03872 | 0.6215 | 0.806 | |
| | Gestational age | 1 | 0.03628 | 0.01656 | 0.5315 | 0.795 | |
| | Birthweight | 1 | 0.02462 | 0.01123 | 0.3606 | 0.911 | |
| | Trial exposure days | 1 | 0.06257 | 0.02855 | 0.9165 | 0.486 | |
| | Residuals | 14 | 0.95579 | 0.43612 | | | |
| | Total | 31 | 2.19156 | 1 | | | |
| DOL 30-60 | Lactoferrin | 1 | 0.04372 | 0.02874 | 0.4859 | 0.783 | |
| | NICU site | 9 | 0.73566 | 0.48355 | 0.9086 | 0.591 | |
| | Health status | 1 | 0.01476 | 0.0097 | 0.164 | 0.967 | |
| | Feed type | 1 | 0.04823 | 0.0317 | 0.5361 | 0.741 | |
| | Gestational age | 1 | 0.03666 | 0.0241 | 0.4075 | 0.822 | |
| | Birthweight | 1 | 0.03737 | 0.02457 | 0.4154 | 0.818 | |
| | Trial exposure days | 1 | 0.12567 | 0.0826 | 1.3968 | 0.31 | |
| | Residuals | 2 | 0.17994 | 0.11827 | | | |
| | Total | 18 | 1.52138 | 1 | | | |

**Metabolites (s)** *(Canberra dissimilarity)*

| DOL | Variable | df | SumOfSqs | R2 | F | Pr(>F) | |
|---|---|---|---|---|---|---|---|
| DOL 0-6 | Lactoferrin | 1 | 0.14918 | 0.11608 | 1.2158 | 0.265 | |
| | NICU site | 4 | 0.52965 | 0.41214 | 1.2 | 0.355 | |
| | Health status | 1 | 0.10832 | 0.08429 | 0.9817 | 0.491 | |
| | Feed type | 1 | 0.10191 | 0.0793 | 0.9235 | 0.539 | |
| | Gestational age | 1 | 0.06577 | 0.05118 | 0.596 | 0.856 | |
| | Birthweight | 1 | 0.07267 | 0.05655 | 0.6586 | 0.797 | |
| | Trial exposure days | 1 | 0.07353 | 0.05721 | 0.6663 | 0.774 | |
| | Residuals | 1 | 0.11035 | 0.08586 | | | |
| | Total | 11 | 1.21138 | 1 | | | |
| DOL 7-9 | Lactoferrin | 1 | 0.10195 | 0.05703 | 0.6545 | 0.821 | |
| | NICU site | 5 | 0.50321 | 0.28152 | 0.6462 | 0.937 | |
| | Health status | 1 | 0.10875 | 0.06084 | 0.6982 | 0.796 | |
| | Feed type | 2 | 0.14521 | 0.08124 | 0.4662 | 0.987 | |
| | Gestational age | 1 | 0.11113 | 0.06217 | 0.7135 | 0.781 | |
| | Birthweight | 1 | 0.11548 | 0.06461 | 0.7414 | 0.754 | |
| | Trial exposure days | 1 | 0.09164 | 0.05127 | 0.5884 | 0.881 | |
| | Residuals | 2 | 0.31151 | 0.17427 | | | |
| | Total | 15 | 1.78749 | 1 | | | |
| DOL 10-14 | Lactoferrin | 1 | 0.11122 | 0.03544 | 1.2006 | 0.312 | |
| | NICU site | 8 | 1.23786 | 0.39439 | 1.6703 | 0.007 | ** |
| | Health status | 1 | 0.12678 | 0.04039 | 1.3685 | 0.174 | |
| | Feed type | 3 | 0.38 | 0.12107 | 1.3673 | 0.09 | . |
| | Gestational age | 1 | 0.06998 | 0.0223 | 0.7554 | 0.749 | |
| | Birthweight | 1 | 0.08211 | 0.02616 | 0.8863 | 0.596 | |
| | Trial exposure days | 1 | 0.10408 | 0.03316 | 1.1235 | 0.338 | |
| | Residuals | 7 | 0.64847 | 0.20661 | | | |
| | Total | 23 | 3.13866 | 1 | | | |
| DOL 20-27 | Lactoferrin | 1 | 0.088 | 0.02667 | 1.0681 | 0.333 | |
| | NICU site | 10 | 1.3819 | 0.4189 | 1.6774 | 0.001 | *** |
| | Health status | 1 | 0.1087 | 0.03295 | 1.3195 | 0.195 | |
| | Feed type | 2 | 0.1299 | 0.03937 | 0.7883 | 0.753 | |
| | Gestational age | 1 | 0.0792 | 0.02401 | 0.9614 | 0.466 | |
| | Birthweight | 1 | 0.0956 | 0.02898 | 1.1603 | 0.284 | |
| | Trial exposure days | 1 | 0.1389 | 0.04212 | 1.6866 | 0.074 | . |
| | Residuals | 12 | 0.9886 | 0.29968 | | | |
| | Total | 29 | 3.2989 | 1 | | | |
| DOL 30-60 | Lactoferrin | 1 | 0.0969 | 0.03969 | 1.0019 | 0.426 | |
| | NICU site | 7 | 0.70972 | 0.29066 | 1.0483 | 0.394 | |
| | Health status | 1 | 0.08146 | 0.03336 | 0.8423 | 0.55 | |
| | Feed type | 2 | 0.20154 | 0.08254 | 1.0419 | 0.397 | |
| | Gestational age | 1 | 0.07397 | 0.0303 | 0.7648 | 0.669 | |
| | Birthweight | 1 | 0.08442 | 0.03457 | 0.8728 | 0.53 | |
| | Trial exposure days | 1 | 0.12233 | 0.0501 | 1.2648 | 0.206 | |
| | Residuals | 6 | 0.58031 | 0.23766 | | | |
| | Total | 20 | 2.44176 | 1 | | | |

**Metabolites (u)** *(Canberra dissimilarity)*

| DOL | Variable | df | SumOfSqs | R2 | F | Pr(>F) |
|---|---|---|---|---|---|---|
| DOL 0-6 | Lactoferrin | 1 | 0.03565 | 0.02352 | 0.9559 | 0.489 |
| | NICU site | 8 | 0.55649 | 0.36716 | 1.865 | 0.001 |
| | Health status | 1 | 0.02854 | 0.01883 | 0.7653 | 0.735 |
| | Feed type | 3 | 0.14694 | 0.09695 | 1.3132 | 0.143 |
| | Gestational age | 1 | 0.0411 | 0.02712 | 1.1019 | 0.32 |
| | Birthweight | 1 | 0.03593 | 0.0237 | 0.9633 | 0.451 |
| | Trial exposure days | 1 | 0.04475 | 0.02952 | 1.1997 | 0.252 |
| | Residuals | 8 | 0.29838 | 0.19687 | | |
| | Total | 24 | 1.51567 | 1 | | |
| DOL 7-9 | Lactoferrin | 1 | 0.03986 | 0.04473 | 0.6972 | 0.854 |
| | NICU site | 6 | 0.33804 | 0.3793 | 1.255 | 0.285 |
| | Health status | 1 | 0.04605 | 0.05167 | 0.8054 | 0.674 |
| | Feed type | 2 | 0.07912 | 0.08877 | 0.8812 | 0.648 |
| | Gestational age | 1 | 0.12695 | 0.14244 | 2.8279 | 0.008 |
| | Birthweight | 1 | 0.03344 | 0.03752 | 0.7449 | 0.75 |
| | Trial exposure days | 1 | 0.04074 | 0.04572 | 0.9076 | 0.556 |
| | Residuals | 1 | 0.04489 | 0.05037 | | |
| | Total | 14 | 0.89123 | 1 | | |
| DOL 10-14 | Lactoferrin | 1 | 0.06031 | 0.02624 | 0.9681 | 0.475 |
| | NICU site | 8 | 0.60475 | 0.26311 | 1.2134 | 0.084 |
| | Health status | 1 | 0.09344 | 0.04065 | 1.4998 | 0.069 |
| | Feed type | 3 | 0.22638 | 0.09849 | 1.2113 | 0.143 |
| | Gestational age | 1 | 0.07368 | 0.03205 | 1.1827 | 0.24 |
| | Birthweight | 1 | 0.09646 | 0.04197 | 1.5484 | 0.049 |
| | Trial exposure days | 1 | 0.06493 | 0.02825 | 1.0422 | 0.379 |
| | Residuals | 14 | 0.87216 | 0.37945 | | |
| | Total | 30 | 2.29848 | 1 | | |
| DOL 20-27 | Lactoferrin | 1 | 0.05226 | 0.0194 | 0.9707 | 0.456 |
| | NICU site | 10 | 0.81453 | 0.30236 | 1.5128 | 0.001 |
| | Health status | 1 | 0.05736 | 0.02129 | 1.0653 | 0.365 |
| | Feed type | 2 | 0.13588 | 0.05044 | 1.2618 | 0.109 |
| | Gestational age | 1 | 0.04768 | 0.0177 | 0.8855 | 0.609 |
| | Birthweight | 1 | 0.07441 | 0.02762 | 1.3821 | 0.111 |
| | Trial exposure days | 1 | 0.03765 | 0.01398 | 0.6993 | 0.877 |
| | Residuals | 23 | 1.23835 | 0.45968 | | |
| | Total | 40 | 2.69392 | 1 | | |
| DOL 30-60 | Lactoferrin | 1 | 0.0979 | 0.0498 | 1.3183 | 0.168 |
| | NICU site | 7 | 0.58117 | 0.29563 | 1.118 | 0.206 |
| | Health status | 1 | 0.05004 | 0.02546 | 0.6739 | 0.882 |
| | Feed type | 2 | 0.13109 | 0.06668 | 0.8827 | 0.642 |
| | Gestational age | 1 | 0.07186 | 0.03655 | 0.9677 | 0.485 |
| | Birthweight | 1 | 0.06625 | 0.0337 | 0.8921 | 0.579 |
| | Trial exposure days | 1 | 0.09056 | 0.04607 | 1.2195 | 0.216 |
| | Residuals | 11 | 0.81685 | 0.41551 | | |
| | Total | 25 | 1.96588 | 1 | | |

*Table presents significant associations between each clinical variable recorded and beta diversity measures across each analysis mode utilized per sample. Significant relationships are highlighted with astrices ($P < 0.05$ = *; $P<0.01$ = **; $P<0.001$ = ***)*

*Kruskal-wallis test: Concordant Vs Discordant siblings average sample composition dissimilarity*

| *Bacteria* | | | |
|---|---|---|---|
| | *Sibling sets* | *Median (IQR)* | *P value* |
| *Concordant* | 17 | 0.42 (*0.32 - 0.51*) | 0.23 |
| *Discordant* | 26 | 0.47 (*0.37 - 0.53*) | |
| ***VOCs*** | | | |
| | *Sibling sets* | *Median (IQR)* | *P value* |
| *Concordant* | 7 | 0.28 (*0.25 - 0.30*) | 0.16 |
| *Discordant* | 18 | 0.25 (*0.22 - 0.28*) | |
| ***Metabolites (s)*** | | | |
| | *Sibling sets* | *Median (IQR)* | *P value* |
| *Concordant* | 1 | 0.26 | 0.82 |
| *Discordant* | 14 | 0.28 (*0.20 - 0.30*) | |
| ***Metabolites (u)*** | | | |
| | *Sibling sets* | *Median (IQR)* | *P value* |
| *Concordant* | 1 | 1.91 | 0.3 |
| *Discordant* | 16 | 0.23 (*0.20 - 0.24*) | |

*No significant differences observed between within group sample compositions of sibling groups who were concordant or discordant for lactoferrin supplementation.*

*Sitewise MaAsLin results: Number of Significantly discriminant features between lactoferrin and placebo samples in each analysis mode*

| | | ***Lactoferrin*** | ***Placebo*** | | | |
|---|---|---|---|---|---|---|
| ***Discriminant features*** | | | | | | ***No discriminant features*** |
| ***Bacteria*** | | | | | | |
| | Single Site | 9 | 4 | | Sites | G, F, C |
| | >1 Site | 5 | 1 | | | |
| ***VOCs*** | | | | | | |
| | Single Site | - | 4 | | Sites | A, B, C, D, E, F, H, I, J, P, S |
| | >1 Site | - | - | | | |
| ***Metabolites (s)*** | | | | | | |
| | Single Site | 736 | 856 | | Sites | B, C, F, H, J, K |
| | >1 Site | 35 | 37 | | | |
| ***Metabolites (u)*** | | | | | | |
| | Single Site | 195 | 126 | | Sites | B, C, D, F, H, J |
| | >1 Site | - | - | | | |

*Features of each analysis mode identified as significantly discriminate between samples from infants receiving lactoferrin and placebo.*

*Merging of positive and negative LCMS ion modes does not impact results*

| | ANALYSIS MODE | | |
|---|---|---|---|
| | **MSCombine** | **Independent** | |
| Total features | 15364 | 17655 | 12% reduction in MSCombine |
| **Stool** | | | |
| Features | 7457 | 5263(+) l 3510(-) | 15 % reduction in MSCombine |
| Median Lactoferrin Richness (IQR) | 5232 (741.8) | + 3684 (538) l - 2426 (364) | |
| Median Placebo Richness (IQR) | 5158 (567.5) | + 3673 (493) l - 2441 (348) | |
| Diff features (Lactoferrin vs Placebo) | 34 (0.46%) | 72 (0.82%) | 0.36% reduction in MSCombine |
| **Urine** | | | |
| Features | 7907 | 4908(+) l 3974(-) | 11% reduction in MSCombine |
| Avg Lactoferrin Richness | 6183.5 (801.0) | + 3889 (498) l - 3058 (390) | |
| Avg Placebo Richness | 6183.0 (741.5) | + 3898 (485) l - 3105 (310) | |
| Diff features (Lactoferrin vs Placebo) | 187 (2.36%) | 285 (3.21%) | 0.85% reduction in MSCombine |

| **METABOLITE COMPOSITION** | *Lactoferrin* | *NICU site* | *Health status* |
|---|---|---|---|
| **Stool combine** | N/A | sig from DOL 10-14 onwards (mean R2 = 0.45) | P = 0.044 @ DOL 10-14 |
| **Stool pos** | N/A | sig from DOL 7-9 onwards (mean R2 = 0.45) | N/A |
| **Stool neg** | N/A | sig from DOL 10-14 onwards (mean R2 = 0.45) | N/A |
| **Urine combine** | P ~ 0.05 @ DOL 0-6 | P<0.001 @ DOL 0-6 & 20-27 | N/A |
| **Urine pos** | P ~ 0.05 @ DOL 0-6 | P<0.001 @ DOL 0-6 & 20-27 | N/A |
| **Urine neg** | P ~ 0.05 @ DOL 0-6 | P<0.001 @ DOL 0-6 & 20-27 | N/A |



Young G, *et al. Arch Dis Child Fetal Neonatal Ed* 2023; 108:272–279. doi: 10.1136/archdischild-2022-324477
